# Cross-Domain Benchmarking of Focus Measures for Smear-Microscopy Autofocus Under a Consensus-Audited Reference

**DOI:** 10.3390/jimaging12090434

**Published:** 2026-09-10

**Authors:** Dineth Hewavitharana, Dumith Jayathilaka, Palitha Dassanayake, Ranjith Amarasinghe

**Affiliations:** Department of Mechanical Engineering, Faculty of Engineering, University of Moratuwa, Katubedda 10400, Sri Lanka; hewavitharanadc.24@uom.lk (D.H.); palitha@uom.lk (P.D.); ranama@uom.lk (R.A.)

**Keywords:** autofocus, focus measure, smear microscopy, digital pathology, cross-domain benchmarking, consensus-referenced evaluation, evaluation sensitivity, computational cost

## Abstract

Focus-measure recommendations for smear microscopy are typically established on one specimen type under one reference definition and one scoring rule, leaving their transferability untested. We benchmark 32 handcrafted focus measures on 26,100 z-stacks spanning five smear-microscopy domains at native acquisition dimensions under one frozen protocol. A fixed ten-voter plurality consensus (REF-B) supplies an identical reference target for every candidate, and two further constructions, single-voter exclusion (REF-A) and a non-derivative four-voter consensus (REF-C), quantify how far the recommendation depends on that target. Ten criteria covering consensus localization, curve shape, perturbation response and kernel cost form a declared cross-domain score; clustered bootstrap resampling, alternative aggregation, weight perturbation and controlled image resampling separate sampling variability from sensitivity to evaluation policy. Gradient responses lead under every analysis. Variance of Gradient ranks first under the primary policy in all 1000 clustered bootstrap replicates and in 67.9% of 1000 sampled weight configurations, while Brenner Gradient attains the lowest consensus deviation, 0.072 planes, at the lowest measured kernel cost. Localization ordering is preserved exactly when the reference is rebuilt from non-derivative voters alone (Spearman ρ = 1.000), although the reference plane itself shifts substantially. The benchmark gives a reproducible, auditable basis for selecting focus measures under stated reference, scoring and image-sampling conditions.

## 1. Introduction

In image-based smear-microscopy autofocus, a focus measure assigns a sharpness response to each acquired plane, and the response maximum identifies a candidate focal plane [1,2]. The same operation supports retrospective selection from a completed z-stack and can form part of an acquisition-time focus search. Choosing the measure is not straightforward because specimen appearance and imaging conditions vary substantially between applications. Usefulness also depends on more than the location of the maximum: the peak should be distinct, competing maxima limited, the response stable under image perturbations, and the computational cost appropriate for the intended workflow. Evaluating these properties together provides more actionable guidance than an isolated ranking under one acquisition condition.

Comparative studies supply much of what is known about focus-measure behavior. Groen and colleagues compared focus functions for cytology; Sun and colleagues evaluated a set of algorithms over a large collection of microscope images; Pertuz and colleagues analyzed focus-measure operators for shape-from-focus under controlled imaging conditions; Nasibov examined hyperspectral microscopy; and Piao and colleagues developed quantitative focus-curve descriptors for optical microscopy [3,4,5,6,7]. Learning-based autofocus can instead estimate axial position directly and performs well within its training regime [8,9,10], but training typically requires suitable focus labels or annotated image sets that are unavailable for many public smear collections. Handcrafted operators therefore remain relevant in their own right rather than only as a fallback: they are training-free, transparent, inexpensive to compute and directly auditable, which matters when an acquisition pipeline must be justified rather than merely tuned.

Smear specimens complicate this comparison in a specific way. Cellular density, stain distribution and spatial architecture vary within and between preparations, and they vary independently of defocus. Quantitative digital pathology makes the same point from the other direction: SpatialQPFs characterize cell-to-cell spatial relationships from segmented cells, and entropy-based descriptors distinguish distributional from spatially aware measures of tissue complexity [11,12]. Those descriptors are not autofocus methods and are not used as such here. They establish that specimen content alone can shift image statistics, which is precisely why a texture or entropy operator may rank well on one preparation and poorly on another, and why a benchmark confined to one specimen distribution cannot settle the question of transfer. 

A ranking under one specimen distribution, reference definition and scoring scheme does not by itself establish suitability across heterogeneous smear-microscopy domains. When an independent focus label is unavailable, an operator-derived reference can favor responses resembling those used to construct it. Criterion weighting introduces a separate choice: prioritizing localization, curve shape or cost can select different operators. A practical benchmark should therefore distinguish sensitivity to the sampled data from sensitivity to how performance is defined and aggregated. The question addressed here is which operator-level and family-level conclusions persist across domains and evaluation choices and which depend on the reference, weighting, aggregation or image-sampling conditions.

**Figure 1:**
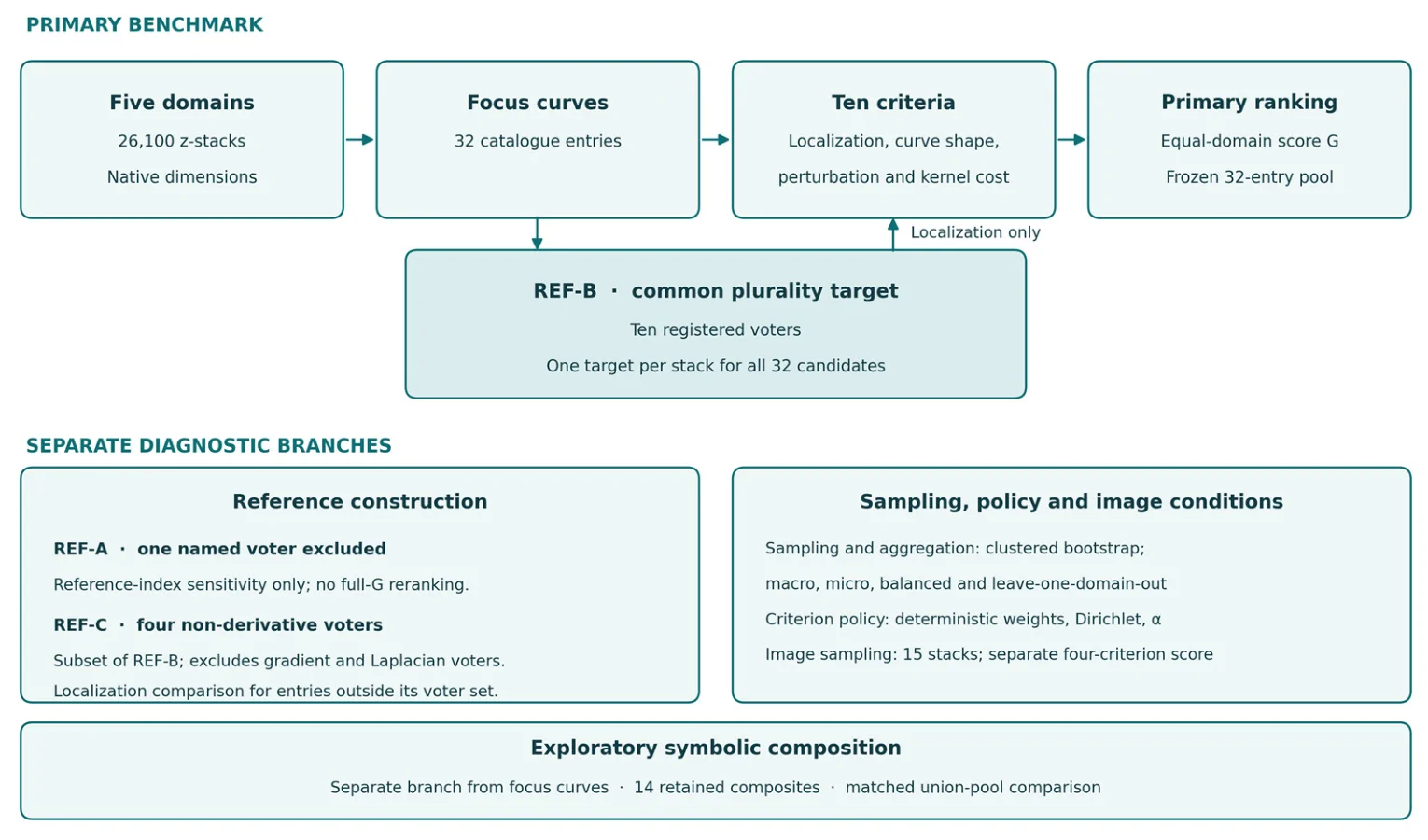
Study workflow. The primary benchmark evaluates 32 catalogue entries at native resolution using ten criteria and an equal-domain score. REF-B provides the common target for localization; curve-shape and cost criteria are computed separately. REF-A measures the sensitivity of reference indices to excluding one named voter. REF-C compares localization ordering for the entries outside its voter set after restricting the consensus to four non-derivative entries. Other branches examine sampling, aggregation, criterion policy and image sampling. Exploratory symbolic composites are compared in a separate matched pool and do not enter the primary ranking.

We address this question with a fixed library of 32 catalogued focus measures evaluated on 26,100 z-stacks from five smear-microscopy domains under a common native-resolution protocol (Figure 1). A fixed ten-voter plurality consensus supplies the same reference target for every candidate. Two diagnostics probe its construction: leave-one-voter-out reference-index sensitivity and localization comparison against a four-voter consensus that excludes gradient and Laplacian voters. Ten criteria combine consensus agreement, focus-curve shape, perturbation response and computational cost. Domain-specific summaries, alternative aggregations, clustered bootstrap resampling, criterion-weight perturbation and controlled image resampling distinguish different sources of variation. An exploratory symbolic-composition analysis asks whether interpretable combinations improve on the strongest individual measure under matched scoring [13,14,15,16,17].

Preliminary comparisons on subsets of these domains were reported previously [18,19]. The present study extends this line of work to a frozen 32-entry library and five-domain native-resolution evaluation, with explicit reference diagnostics and separation of sampling uncertainty from scoring-policy sensitivity. Its contribution is the integrated evaluation protocol and the interpretation of operator recommendations across these choices. This study evaluates the focus-measure component retrospectively from acquired z-stacks; it does not evaluate a complete autofocus controller.

## 2. Materials and Methods

### 2.1. Study Design

The primary benchmark computes focus curves for all 32 catalogue entries, evaluates ten criteria and forms an equal-domain aggregate score (Figure 1). REF-B supplies one fixed target per stack for the localization criterion. REF-A examines reference-index changes after excluding one named voter; REF-C compares localization ordering for the entries outside its voter set. Neither diagnostic is presented as a full ten-criterion reranking. Sampling, aggregation, weighting and image-resampling analyses address separate questions. Symbolic composition is exploratory and does not enter the primary ranking. Analyses use Python 3.10 with NumPy 1.26.4, OpenCV 4.11.0.86, SciPy 1.12.0, PyWavelets 1.6.0 and DEAP 1.4.3; Table S23 inventories the machine-readable release and identifies the file supporting each reported analysis.

### 2.2. Datasets and Acquisition Metadata

Five domains from three public resources were evaluated (Table 1). The white blood cell (WBC) domain contains 25,773 ten-plane, 200 × 200-pixel crops from a source cohort of 72 patients and 214 slides, acquired at a 0.4 µm axial step and 50× magnification [20]. The benchmark mapping links crops to slides but not slides to individual patients. Peripheral blood smear (PBS), bone marrow aspirate (BMA) and thick blood film (TBF) stacks have released dimensions of 2160 × 2560 pixels, a 0.5 µm step and a 100×/1.4 NA oil-immersion objective [21,22]. The TbImages tuberculosis-sputum domain (TBI) contains 30 ten-plane stacks of 2112 × 2816 pixels, acquired at a 2.5 µm step with a 100×/1.25 NA oil-immersion objective [23,24]. The primary analysis preserves the released dimensions without additional resizing or region-of-interest cropping. Pixel size is not consistently reported and was not inferred.

Axial sampling is not uniform, so a plane is not a common physical unit across domains. Localization is reported within each domain in planes and micrometers using its documented step. Curve-shape criteria expressed in planes are normalized only within a domain before cross-domain scoring; this aligns numerical scales but does not remove differences in axial sampling or optical resolution. WBC supplies 98.7% of stacks and 98.3% of planes, motivating the separate aggregation estimands described below.

**Figure 2:**
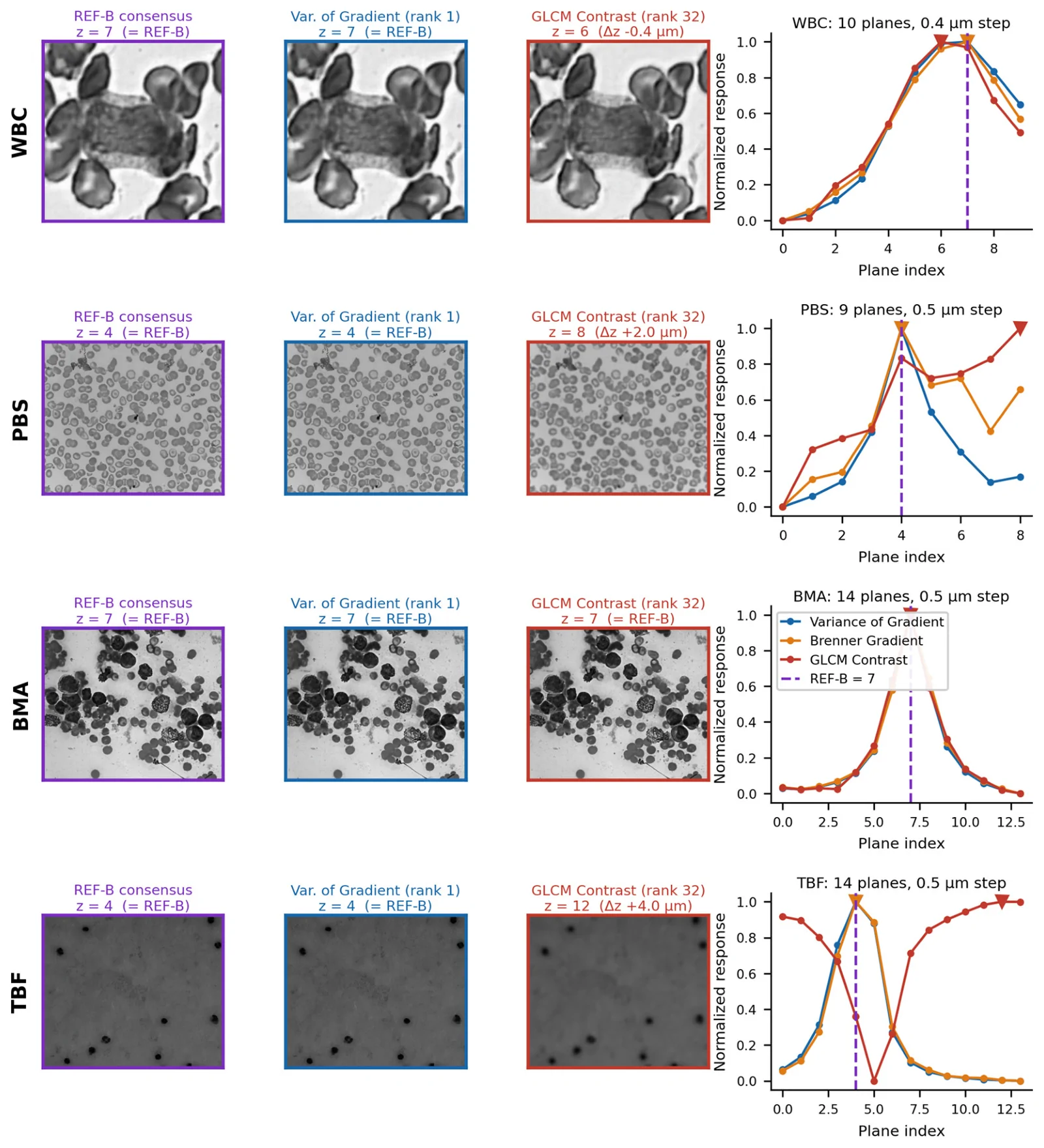
Representative stacks from WBC, PBS, BMA and TBF. The first three columns show planes selected by REF-B, Variance of Gradient and GLCM Contrast, respectively. The fourth shows focus curves with selected indices marked by triangles and REF-B by a dashed line. Indices are zero-based. Stack selection and display normalization are described in Section 2.2. These examples illustrate the data and operator selections, not independent focus validation. Triangles mark the plane selected by each operator and the dashed line marks the REF-B reference index.

Figure 2 illustrates four domains with documented permission for image reproduction. One stack per domain was selected as the first stack in deterministic source order, without visual screening. All displayed planes within a stack use the same intensity minimum and maximum. The selections are from REF-B, Variance of Gradient and GLCM Contrast, the latter two representing the highest- and lowest-ranked entries in the completed primary analysis. Indices were obtained from prediction outputs rather than assigned by visual assessment. These examples illustrate agreement and disagreement, not independent optical-focus labels.

### 2.3. Focus-Measure Library and Curve Computation

The 32 operators span gradient, Laplacian, statistical, entropy, texture, frequency and wavelet-labelled families [25]; operator provenance is listed in Table A1, and Table S1 gives the same catalogue with each entry’s implementation-correspondence group, defined in Table S2, and its reference membership. Each operator returns one scalar per plane. The source encoding range is identified before floating-point conversion and mapped consistently to [0, 1], and Histogram Entropy is binned into 256 fixed bins over that encoding range. Binning on the stored range rather than on post-conversion values is necessary because a uint16 source would otherwise contribute only its lowest 256 levels; eleven dtype and regression tests enforce this behavior, and Supplementary Section S3 reports the effect of the convention on each domain (Table S3 and Figure S1). 

A small number of catalogue entries share a common underlying implementation and therefore return the same focus response. These correspondences were established by comparing the raw focus curves of every pair of entries across all 26,100 stacks and are documented in the Supplementary Materials (Table S2). All entries are retained separately so that the frozen pool matches published operator lists.

Within each stack, the curve f of operator *j* on stack *s* is min–max normalized to [0, 1] before the curve-shape criteria are calculated (Equation (1); subscripts *j* and *s* are suppressed there for legibility). Constant curves are mapped to zero. The evaluated operator’s predicted focal index is the global maximum over the whole curve, including the two endpoint planes, with ties resolved by the upper central index among the sorted tied indices. This differs deliberately from the voter rule in Section 2.4, which excludes endpoints; the consequence is quantified there. Source filenames are ordered by natural numeric sort. Internal indices are zero-based; reported displacement is unchanged by index origin.

(1) $$\tilde{f}(z)=\frac{f(z)-min\;f}{max\;f-min\;f}$$

### 2.4. Reference-Focus Construction and Sensitivity

An independent hardware-calibrated or expert-labeled focal index is not available across all five resources. The reference is therefore algorithmic: a plurality vote over per-voter peak indices, not an arithmetic mean of normalized curves. For voter *m* and stack *s*, let *p_m_,_s_* denote its selected index. Equation (2) counts votes at each plane, and Equation (3) applies the tie-breaking function *T* to the indices with the highest count.

(2) $$N_{s}(z)=\sum_{m\;\in \;V}1(p_{m,\;s}=z)$$

(3) $${z_{B,\;s}}^{*}=T(\underset{z}{argmax}\;N_{s}(z))$$

Three conventions require explicit statement. First, the rule is plurality rather than majority: the selected plane is the one receiving the most votes and need not receive more than half of them. Second, *T* is the implemented tie-break. It sorts the tied indices and returns the middle element, taking the upper–middle element when the number of tied indices is even. *T* therefore operates on the tied vote labels and does not necessarily return the plane nearest the physical center of the stack. The same rule resolves ties inside an individual voter curve. Third, voter eligibility excludes the two endpoint planes whenever a stack contains at least three planes, so no reference index can fall on the first or last acquired plane. One reference is constructed per stack and per preprocessing condition.

The endpoint convention is asymmetric: an evaluated candidate may select an endpoint that the reference cannot occupy. Across all 32 entries and 26,100 stacks, 9.0% of predictions fall on an endpoint, ranging from 4.7% in BMA to 35.5% in TBF. The rate is 0.20–0.34% for the six leading catalogue entries. Endpoint predictions are therefore uncommon among those entries, but the convention can penalize boundary-peaking alternatives, and its effect on relative family comparisons is not isolated by these rates.

REF-B applies the same target to all 32 candidates. Its registry *V* contains Tenengrad, Brenner Gradient, Energy of Gradient, Variance of Gradient, Variance of Laplacian, Sum-Modified Laplacian, Normalized Variance, Histogram Entropy, GLCM Contrast and Fourier-Transform Sharpness Index (Table 2 and Table A1). The executed registry is retained for traceability. REF-B therefore includes direct self-voting and correlated families; it is a common diagnostic target, not an independent optical standard.

REF-A excludes the evaluated entry *j* from *V* when *j* is a registered voter; otherwise it retains V. Let *V_j_* denote this set and *N_s_,_j_* its vote count, formed as in Equation (2). Equation (4) applies the same plurality and tie-breaking rules. A registered voter is compared with a nine-entry target, whereas a non-voter retains REF-B. This removes the named entry’s direct vote, but an identical or related response can remain. REF-A is used here to quantify reference-index sensitivity to a single exclusion, not to establish an independent target or a full-score rank comparison.

(4) $${z_{A,\;s,\;j}}^{*}=T(\underset{z}{argmax}\;N_{s,\;j}(z))$$

REF-C is a family-shift diagnostic using the four-entry set *V꜀*: Normalized Variance, Histogram Entropy, GLCM Contrast and Fourier-Transform Sharpness Index. This is a subset of the REF-B registry containing no gradient or Laplacian entries, not a wholly independent reference source. REF-C uses the same plurality, endpoint and tie-breaking rules with *V* replaced by *V꜀*. Its localization comparison covers the entries outside *V꜀*; the four reference voters are excluded.

REF-C tests whether localization ordering persists when the gradient and Laplacian voters are removed. It is not assumed to be a more accurate reference: disagreement with REF-B measures sensitivity to voter composition, not the error of either reference relative to optical best focus. The analysis compares equal-domain mean absolute displacement under REF-B and REF-C for the same set of entries. It does not recompute the full ten-criterion score G under REF-C. Full per-voter and per-domain diagnostics for all three constructions are reported in Supplementary Section S5.

The TBI source describes an acquisition focus convention, but the released ordering metadata do not establish an independent label for every distributed stack, so no external reference was constructed for that domain [23].

### 2.5. Evaluation Criteria and Decision Score

Ten criteria describe consensus localization, near-peak range, false maxima, full width at half maximum, curve roughness, steep-to-gradual slope ratio, steep-slope width, peak curvature, response to additive image noise, and operator-kernel runtime (Table 3 and Table A2). Raw criterion values are directionally aligned so that lower is better. Within each domain–criterion cell, Equation (5) normalizes the aligned value a across the frozen 32-entry pool C; the cell minimum and range Δ are computed over that pool. A constant cell is assigned zero. This across-candidate normalization is distinct from within-stack normalization in Equation (1). G is therefore a candidate-pool-relative decision score, not an absolute performance scale: adding or removing candidates can change it. Raw values should be read alongside normalized scores when comparing individual criteria.

The criteria are drawn from three sources rather than proposed here. Absolute peak-localization error, false-maxima count and full width at half maximum follow classical autofocus evaluation practice [3,4,26]. The steep-to-gradual slope ratio, steep-slope width, curvature at peak and the relative response change under an additive perturbation follow the focus-curve descriptors of Piao and colleagues [7], adapted here to discrete stacks of nine to fifteen planes and to one fixed perturbation model. Range around the global maximum, focus-curve roughness and execution time per plane are implementation-oriented criteria added for this benchmark: the first two describe plateau and smoothness behavior that affects which plane is selected, and the third makes computational cost an explicit component of the comparison rather than a postscript. The contribution is the combination and the declared scoring policy, not the individual definitions.

(5) $$x_{j,\;d,\;k}=\frac{a_{j,\;d,\;k}-{a_{d,\;k}}^{min}}{\Delta_{d,\;k}}$$

(6) $$S_{j,\;d}=\sum_{k}w_{k}x_{j,\;d,\;k}$$

(7) $$\mu_{j}=\frac{1}{5}\sum_{d}S_{j,\;d}$$

(8) $$\sigma_{j}=\sqrt{\frac{1}{5}\sum_{d}{\left(S_{j,\;d}-\mu_{j}\right)}^{2}}$$

(9) $$G_{j}=\alpha \;\mu_{j}+(1-\alpha )\;\sigma_{j}$$

Equations (6)–(9) define the domain scores, their mean *μ*, population standard deviation *σ* across D = 5 observed domains (denominator D), and combined score *G*. Lower *S* and *G* are preferred. The primary α = 0.7 prioritizes a low cross-domain mean while retaining a penalty for variability; low dispersion alone can reward consistently poor performance. The criterion weights in Table 3 give the greatest emphasis to localization and near-peak range while retaining curve-shape and computational-cost considerations; Table S4 lists the same weights with the internal identifier used in the machine-readable release. Both *α* and the weights are author-defined engineering choices, not expert-elicited or data-optimized values, and are not claimed to be optimal or prospectively prespecified. Sensitivity analyses vary *α* (Table S5) and use alternative deterministic weights (Table S6) and 1000 draws from Dirichlet(1, …, 1), whose ten non-negative weights sum to one. The random draws explore scoring priorities; their winner frequencies are not sampling confidence probabilities.

### 2.6. Aggregation and Uncertainty

The ten criteria are related properties of the same focus curves, not independent experimental replicates. Sampling uncertainty is therefore assessed by 1000 paired clustered bootstrap replicates [27], using the same sampled units across entries and recomputing criteria, domain scores, *G* and ranks. WBC is clustered by its 214 slides; slide-to-patient linkage for the 72-patient source cohort is unavailable. The other domains are resampled by stack because no higher-level grouping identifier is released. Unrecorded dependence between stacks cannot be removed by this design. No inference treats the 50 domain–criterion cells as independent blocks.

Four estimands are kept separate: domain-specific performance; equal-domain macro aggregation (primary); per-stack microaggregation, which describes the observed and WBC-dominated corpus; and 1000 balanced subsamples of 30 stacks per domain. Leave-one-domain-out analysis describes dependence on the five observed domains. These estimands answer different questions and are not treated as interchangeable estimates of one population quantity. Sensitivity of the reference index itself is treated as a separate source of uncertainty: per-voter and per-domain results for REF-A, and the agreement and localization-ordering comparisons between REF-B and REF-C, are reported in Tables S7, S8 and S9 and Figures S2, S3 and S4.

### 2.7. Computational-Cost Protocol

Runtime was measured on one x86-64 workstation in Python 3.12 under the recorded software environment. Three deterministic images per domain were evaluated after two warm-ups with seven repeats; the domain–resolution–operator job order was randomized. OMP, OpenBLAS, MKL and NumExpr thread environment variables were set to one, while the recorded OpenCV runtime reported 32 threads; the protocol is therefore not described as fully single-threaded. The main comparison uses per-plane operator-kernel time; I/O, preprocessing and combined timings are provided in Supplementary Section S9. For each operator, the equal-domain macro statistic is the arithmetic mean of the five domain-specific median kernel times. The corpus-weighted microstatistic is the slice-count-weighted mean of those same five domain medians. These summaries compare computational cost only and exclude exposure, transfer, stage motion, settling, search and controller overhead.

### 2.8. Exploratory Resampling Sensitivity

A controlled 15-stack experiment varies aspect-preserving scale, interpolation kernel and forced-square distortion. Each domain contributes its first, middle and last stack in deterministic order. The frozen library is evaluated under 14 conditions after mapping each image to an aspect-preserving base. Within this experiment, a descriptive four-criterion score combines peak error, false maxima, curve roughness and full width at half maximum. It is distinct from the full-corpus ten-criterion score G. Changes in these descriptors are compared with rank shifts; Spearman associations are exploratory, not causal estimates. Full per-condition results are reported in Supplementary Section S9.

### 2.9. Exploratory Symbolic Composite Search

Strongly typed genetic programming was run in five leave-one-domain-out folds and one all-domain refit. In each fold, ten terminals were selected using training-domain results only, with family stratification and at most two terminals per family. Ten seeds used a population of 500, 100 generations, protected arithmetic primitives, a maximum depth of 10, and a maximum size of 35 nodes [13,14,15,16,17,28,29].

Evolution used only training domains, but post-evolution processing was not fully held out. Exact duplicates were collapsed, and same-fold representatives were deduplicated by correlation of their mean held-out curves, then ordered by held-out score, complexity and seed. The resulting 14 retained composites therefore include held-out information in representative selection. Fold scores are reported as descriptive transfer summaries, not unbiased validation estimates. The final comparison of all retained composites and single operators uses one matched full-data union pool because the normalization ranges depend on the candidates present. Provenance for every retained composite is reported in Supplementary Section S10.

## 3. Results

### 3.1. Operator Ranking and Localization

Under REF-B, equal-domain aggregation and α = 0.7, Variance of Gradient attains the lowest score (G = 0.1058; Table 4); the complete ranking of all 32 entries is given in Table S10 and their domain scores in Table S11. Tenengrad, Squared Gradient, Energy of Gradient and Gradient Squared Energy follow at G = 0.1103 to 0.1105, separated by measured kernel time, which ranges from 21.2 ms per plane for Tenengrad to 24.0 ms for Gradient Squared Energy. Brenner Gradient follows at G = 0.1143. The leading tier is therefore occupied entirely by gradient operators, and these entries provide the starting point for the sensitivity analyses.

**Figure 3:**
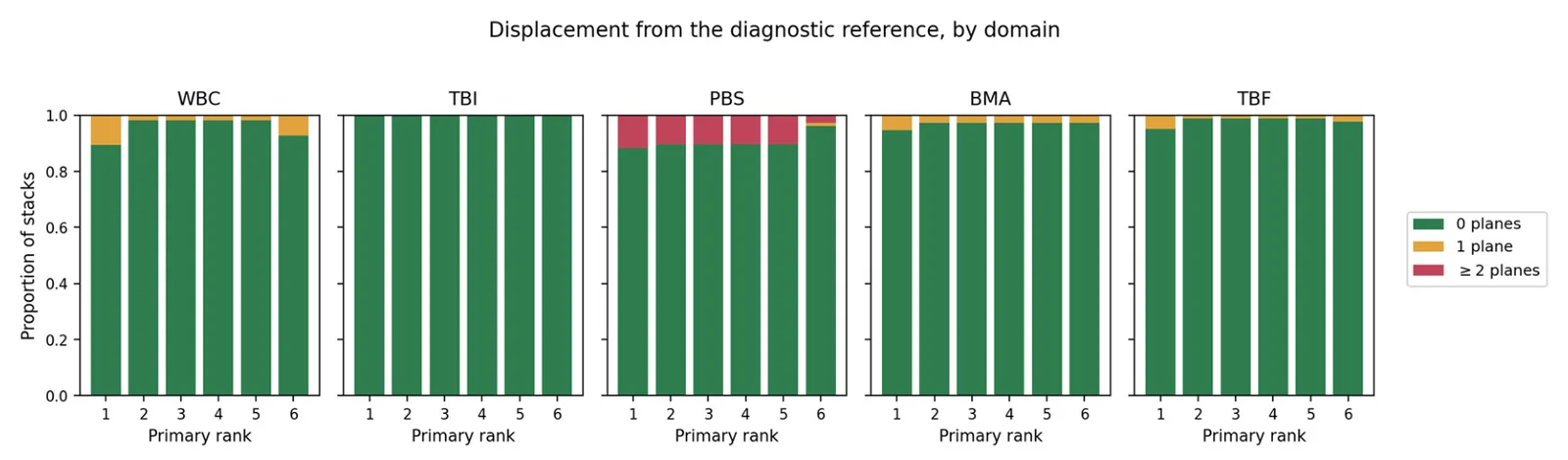
Displacement from REF-B by domain for the six leading catalogue ranks under the primary policy. Categories indicate exact agreement, displacement by one plane, and displacement by two or more planes; they describe agreement with the consensus target.

For Variance of Gradient, mean deviation from REF-B is 0.111 planes on WBC, 0 on TBI, 0.519 on PBS, 0.053 on BMA and 0.049 on TBF, corresponding to 0.044, 0, 0.260, 0.026 and 0.025 µm under each domain’s documented axial step; per-domain values for the leading entries are given in Supplementary Table S12. These fractional values are averages of integer plane selections and do not imply sub-plane estimation. Exact-match rates are 89.3%, 100%, 88.3%, 94.7% and 95.1%, and within-one-plane rates are 99.8%, 100%, 88.3%, 100% and 100% (Figure 3). PBS is the hardest domain for every leading operator: its within-one-plane rate equals its exact rate, so the residual errors there are displacements of two planes or more rather than near misses.

The lowest aggregate score is not the lowest localization error, and Table 5 makes that visible: Brenner Gradient attains a smaller equal-domain mean deviation (0.072 planes) than Variance of Gradient (0.146) while scoring worse overall, because the aggregate also weights peak-range control, curve shape and kernel cost. Readers whose application is dominated by localization alone should therefore read the deviation column rather than G; raw, directionally unaligned values of the four highest-weighted curve criteria for all 32 entries are given in Table S13.

### 3.2. Sensitivity of the Reference

REF-B supplies one identical target for every candidate. Because a non-voter retains the complete ten-voter set, REF-A differs from REF-B only for the ten voters. Excluding one voter leaves the plurality outcome unchanged in 96.0–99.9% of WBC stacks, 94.8–98.7% of PBS stacks and 95.6–100% of TBF stacks, and changes nothing on TBI or BMA; mean absolute displacement is at most 0.041 planes on WBC, 0.156 on PBS and 0.209 on TBF. Per-voter values for every domain are tabulated in Supplementary Table S7. These figures characterize the reference index alone. A matched recalculation of the full ten-criterion score under candidate-specific REF-A targets was not generated, so index agreement is not evidence that operator rankings are unchanged.

Reference indices occupy planes 1–8 of the ten-plane WBC stacks and never an endpoint. Evaluated candidates select endpoints in 9.0% of all entry–stack pairs, compared with 0.20–0.34% for the first six catalogue entries. These frequencies document the asymmetry but do not isolate its contribution to the performance gap between families.

Changing the voter composition substantially changes the reference plane (Table 6A). REF-B/REF-C exact agreement is 49.4% on WBC, 71.4% on PBS, 91.8% on TBF, 94.7% on BMA and 100% on TBI. On WBC, the mean displacement is 0.950 planes, and the 90th percentile is two planes. The equal-domain mean deviation of Brenner Gradient rises from 0.072 planes under REF-B to 0.412 under REF-C, and that of Variance of Gradient from 0.146 to 0.511. Absolute localization values are thus specific to the target used.

Despite these target changes, the equal-domain localization ordering of the eligible entries is unchanged: every entry retains its rank, Spearman ρ = 1.000, and top five and top ten membership is identical (Table 6B). Brenner Gradient ranks first under both references, the gradient-energy entries follow, and Variance of Gradient ranks seventh in each case. The ordering is therefore not an artifact of gradient and Laplacian voting in REF-B. The result applies to this mean absolute-displacement metric and the two operator-derived targets and does not by itself establish reference invariance of the full G ranking.

Reference-index agreement and localization-rank agreement summarize different quantities. The former measures how often the target changes; the latter asks whether those changes alter relative mean absolute displacement. In these data, larger deviations under REF-C coexist with an unchanged ordering. This is empirical rank robustness, not a mathematical cancellation property: a common target shift can affect different candidates’ absolute errors differently.

### 3.3. Sampling, Weighting and Aggregation Sensitivity

**Figure 4:**
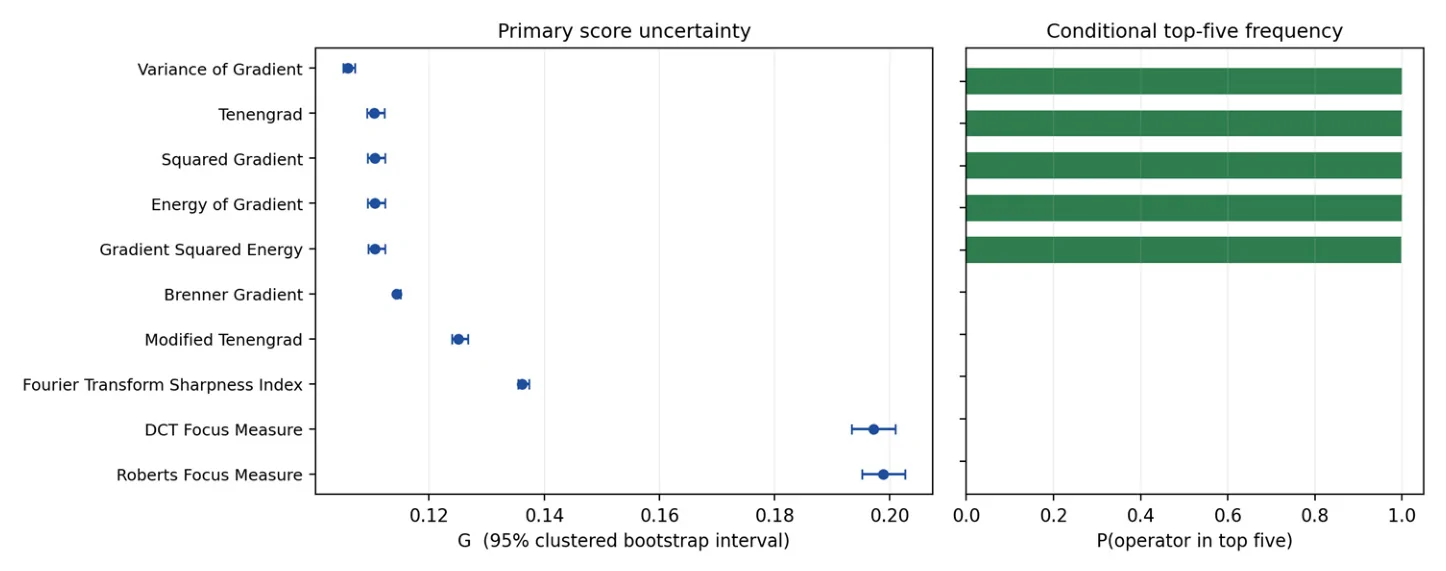
Clustered-bootstrap intervals for G and top five frequencies for catalogue entries under the fixed primary policy, from 1000 paired replicates with WBC clustered by slide. The right panel uses the same entry order as the left. These frequencies describe data-resampling stability, not sensitivity to criterion priorities.

Sampling, scoring policy and domain aggregation address different sources of variation. Under 1000 paired clustered bootstrap replicates, Variance of Gradient ranked first throughout, with a percentile interval for G of 0.1051–0.1071. The catalogue entries occupying the primary top five appeared in the bootstrap top five in at least 99.9% of replicates (Figure 4). Results for all 32 entries are given in Table S14 and Figure S5.

The individual winner changes when criterion priorities change. Across 1000 Dirichlet draws, Variance of Gradient ranked first in 67.9% of configurations, Fourier-Transform Sharpness Index in 13.8%, Brenner Gradient in 4.4% and Intensity Range Index in 3.8% (Table S15 and Figure S6). Varying α from 0 to 1 in steps of 0.1 left Variance of Gradient first, without establishing an optimal α. The random-weight winners include both gradient and non-gradient entries. Applications that prioritize different criteria may therefore select a different operator even when the data are unchanged.

Aggregation changes the estimand rather than merely adding variance. The equal-domain, per-stack, balanced-subsample and leave-one-domain-out analyses all preserve the gradient-leading tier while changing numerical scores and lower ranks (Tables S16 and S17). For Variance of Gradient, mean displacement is 0.146 planes under equal-domain aggregation, 0.111 under the WBC-dominated per-stack estimand, and 0.146 planes across balanced subsamples, with a standard deviation of 0.047 across the 1000 subsamples. Operator-kernel times for every entry, domain and image size are tabulated in Tables S18 and S19 and Figure S7, and are discussed in Section 3.5.

### 3.4. Image-Resampling Sensitivity

**Figure 5:**
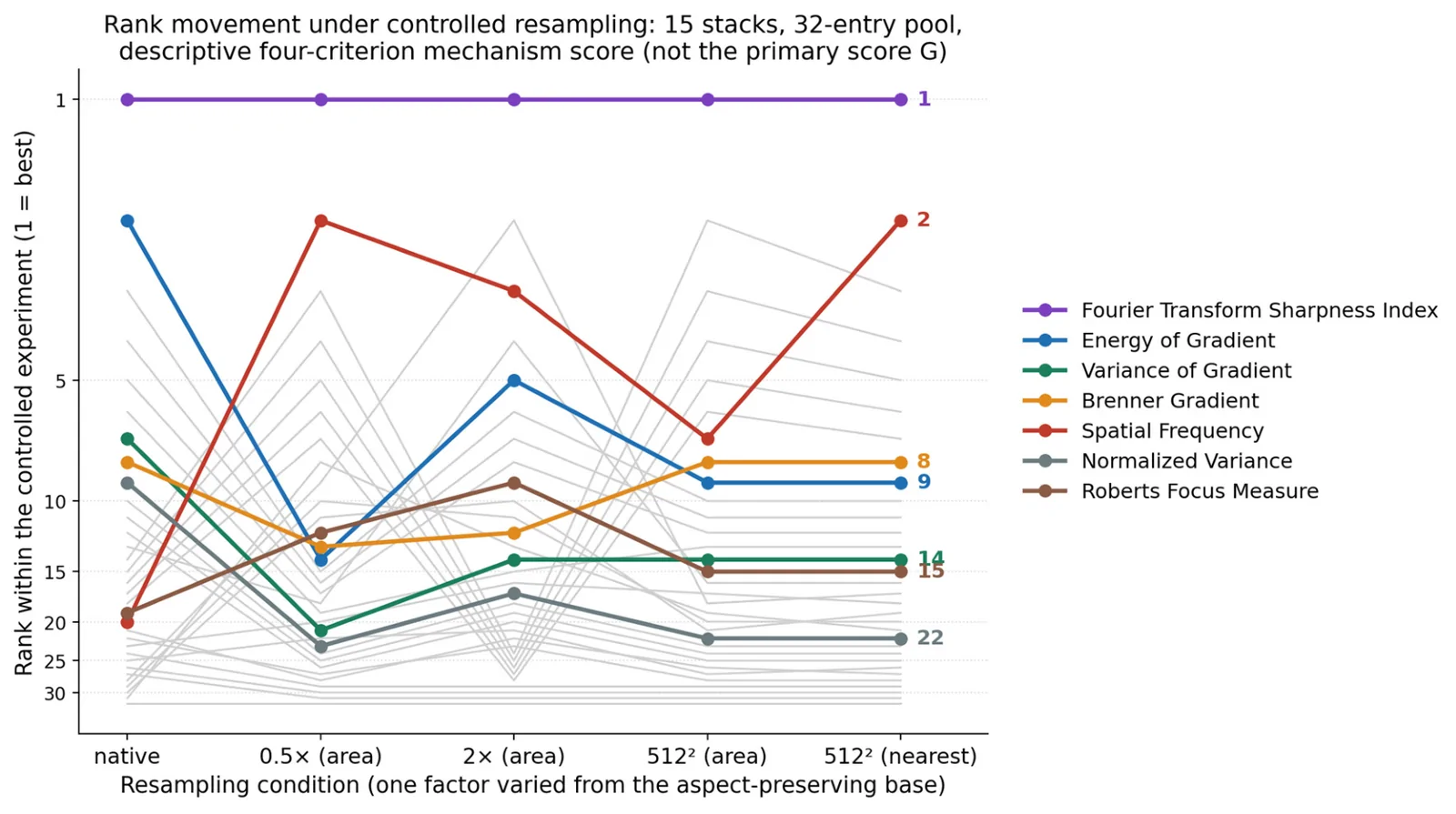
Rank movement in the controlled 15-stack resampling experiment. Ranks use a descriptive four-criterion score within the same 32-entry pool, not the primary ten-criterion score G; only within-experiment shifts are comparable. Grey lines show the remaining catalogue entries, plotted for context and not individually labelled.

Resampling changes rankings substantially (Figure 5). This experiment uses a different and narrower score from the primary ranking, so it cannot be compared with the sensitivity analyses above to identify the largest source of variability; it establishes only that ranks move under image sampling and by how much within its own scoring. Within the controlled experiment, which uses 15 stacks, the same 32-entry pool and a descriptive four-criterion mechanism score rather than the primary score G, Energy of Gradient falls from rank 2 at native resolution to rank 14 under 0.5× area downsampling and rank 9 on a forced 512 × 512 grid, while Spatial Frequency rises from rank 20 to rank 2 and Roberts Focus Measure from rank 19 to rank 12; ranks for all 32 entries under the representative conditions are given in Table S20, and under all 14 conditions in Figure S8. Ranks in this experiment are internal to it, so only within-experiment movement is interpretable.

The associations behind that movement are informative but weak evidence of mechanism. Under 0.5× area downsampling, rank improvement correlates only weakly with reduced curve roughness (ρ = 0.104) and more strongly with fewer false maxima (ρ = 0.582) and narrower full width at half maximum (ρ = 0.526); under 2× area upsampling, the strongest association is with reduced peak-localization error (ρ = 0.676). Short-support operators respond to structures measured in pixels, so changing the sampling grid changes what they respond to. These descriptors also contribute to the four-criterion score, so their correlations with its rank changes contain a part–whole relationship. Together with the 15-stack sample, this limits interpretation to descriptive associations rather than independent evidence of mechanism; the full set of associations by condition is given in Table S21.

### 3.5. Computational Cost

**Figure 6:**
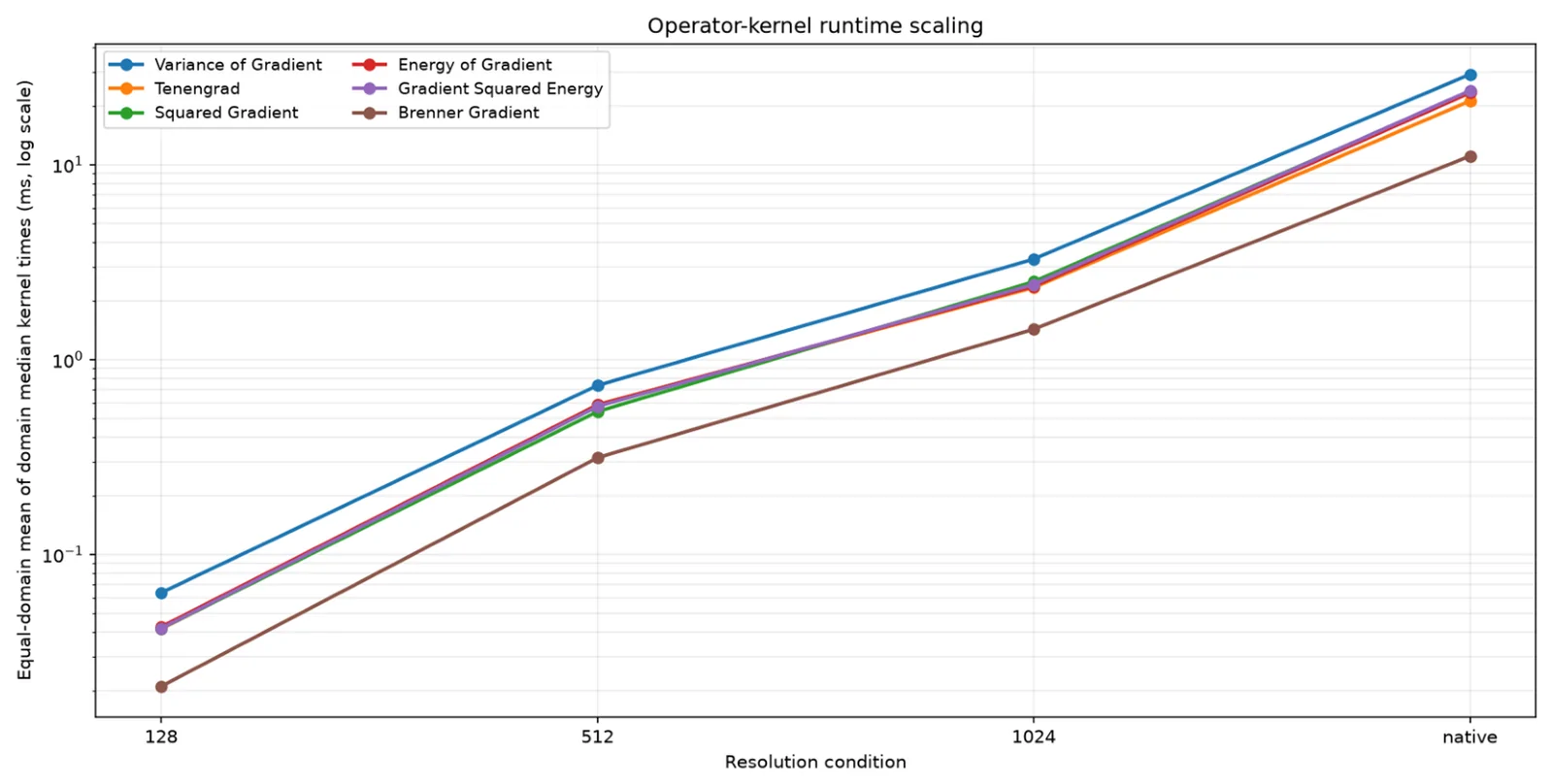
Comparative operator-kernel times for standardized image sizes and the mixed native-resolution workload. Each point is the equal-domain mean of five domain-specific medians under Section 2.7. “Native” combines the different dimensions in Table 1 and is a workload category, not a fourth numerical image size; joining lines do not imply a continuous size trend to that category. Values and ordering describe the recorded implementation and environment, not system-level autofocus latency.

For the six leading gradient catalogue entries, the equal-domain mean of five domain-specific median kernel times ranges from 11.1 ms per plane for Brenner Gradient to 29.1 ms for Variance of Gradient (Table 5; Figure 6). The slice-count-weighted mean of the same domain medians is 0.27–0.72 ms, because the small 200 × 200 WBC planes dominate the observed corpus. Both statistics describe the same implementation under different weightings of the same five domain medians, and both are reported so that corpus composition is visible rather than hidden inside a single average. These are comparative operator costs under the measured protocol; they exclude acquisition and closed-loop control, and no real-time claim follows from them.

### 3.6. Exploratory Symbolic Composites

Fourteen composites were retained under the rule stated in Section 2.9, with provenance for each given in Table S22. In the matched all-domain union pool, the best composite (CFM1, the all-domain refit, 33 nodes and depth 7) reached G = 0.1622 at rank 10, whereas Variance of Gradient reached G = 0.1182 at rank 1. The most compact retained expression, CFM4, uses nine nodes at depth 5 and combines four terminals: Fourier-Transform Sharpness Index minus the product of the Curvelet Transform Sharpness Index entry, a db1 wavelet proxy, with a doubly protected square root of the difference between Brenner Gradient and Gradient Squared Energy. Equivalently, CFM4 = FTSI − CTSI × psqrt (psqrt(Brenner − GSE)), where CTSI is the db1 proxy and psqrt denotes the implemented protected square root. No retained composite improved on the strongest single operator.

The same ordering holds in the descriptive fold summaries (Table 7): in every fold, the lowest retained-composite held-out score exceeds the lowest score among that fold’s selected single terminals by 0.053–0.138. Because held-out curves and scores contributed to post-evolution deduplication and ordering, these values are descriptive transfer summaries rather than unbiased estimates. Even under that favorable selection, they provide no evidence that the evaluated symbolic combinations improve on the strongest selected single operators (Figure S9).

## 4. Discussion

### 4.1. What Transfers Across These Domains, and What Does Not

The most consistent pattern across the analyses is the strength of gradient responses, not a universally best operator. Variance of Gradient leads under the primary score and is stable under clustered data resampling, but other entries win in 32.1% of sampled criterion-weight configurations. Brenner Gradient offers lower mean consensus deviation and lower measured kernel cost, while the gradient-energy entries provide another strong baseline. Their relative merits depend on the criteria emphasized. This complements earlier microscopy comparisons [3,4,5,6,7] by making the reference, specimen balance and scoring choices explicit.

Both criterion reweighting and image resampling changed individual ranks. Their magnitudes cannot be compared as a decomposition of sensitivity because the resampling experiment used 15 selected stacks and a different four-criterion score. The practical implication is to check a recommendation under both the intended scoring priorities and the working image resolution, rather than to regard one source of variability as universally dominant.

### 4.2. Reference Construction and Domain Aggregation

REF-B resolves the unequal-target problem by assigning every entry the same plurality-vote index. REF-A shows that this index usually survives exclusion of one named voter while retaining related or identical responses. REF-C provides a different diagnostic: after removing gradient and Laplacian voters, the reference changes substantially on WBC, but the localization ordering of the entries outside its voter set remains unchanged. These checks assess different aspects of reference construction and should not be interpreted as interchangeable validations.

The localization result is therefore robust across the two tested voter compositions under the chosen equal-domain metric, while its absolute magnitude is reference-dependent. REF-C is a subset of the REF-B registry and shares the same peak-selection, endpoint and tie-breaking conventions. It is not an independent optical standard, nor does unchanged localization ordering establish that the complete G ranking is reference-invariant. Blinded expert focal intervals or an independent optical reference would address a different question: whether agreement with these algorithmic targets corresponds to acceptable physical focus.

Domain imbalance changes the estimand rather than merely inflating variance. The per-stack summary describes the observed corpus and is effectively a WBC result; equal-domain aggregation removes that numerical dominance but grants a 30-stack domain the influence of a 25,773-stack domain, and it does not equalize precision across domains. Balanced subsampling and leave-one-domain-out results show that the gradient-tier conclusion persists under the tested alternative summaries, while the domain-specific values remain the relevant numbers for a deployment decision on a single specimen type.

### 4.3. Image Sampling and Computational Cost

Preprocessing is part of the operating conditions of a focus measure. Roberts and Brenner use short, fixed pixel offsets, so scale and interpolation change the spatial structures represented by those differences. Changes in competing maxima, peak widths and localization offer plausible explanations for rank movement. However, the descriptors contribute to the resampling score itself, and the experiment contains only 15 selected stacks. The findings motivate explicit checks of preprocessing, not a causal or universal rule about which resampling method benefits an operator.

Computational cost is conditional on resolution and on how domains are weighted. The equal-domain and corpus-weighted statistics describe one implementation under two weightings of the same five domain medians, and they differ by roughly a factor of forty purely because of corpus composition. They support a comparative cost criterion for choosing among operators; exposure, stage motion, settling, readout and search logic must be measured in the intended microscope before any latency claim can be made.

### 4.4. Symbolic Composition as a Secondary Analysis

Symbolic composition did not improve on the strongest individual measure under matched full-data scoring. The descriptive fold summaries also favor selected single terminals, although held-out-informed retention prevents an unbiased validation interpretation. This negative result is informative about the tested terminal sets, search budget and scoring regime. It neither rules out more effective symbolic combinations nor establishes that gradient operators are sufficient outside the evaluated conditions.

### 4.5. Specimen Structure and the Interpretation of Texture and Entropy Operators

Entropy and texture operators should be read in the context of specimen structure. Cellular density, stain distribution and architecture alter gray-level distributions and co-occurrence independently of defocus [11,12], which is a plausible reason why Histogram Entropy and GLCM Contrast rank in the lower half of this library on some domains and not others. The operators evaluated here are global pixel-level statistics: Histogram Entropy is a gray-level Shannon entropy, and GLCM Contrast is a local co-occurrence statistic, and neither represents segmented-cell spatial organization. Cell-level spatial descriptors are a distinct class of measurement and were outside this study; relating focus responses to cell-level organization would require a separate analysis.

### 4.6. Relation to Prior Benchmarks

Focus-measure comparisons have been reported for several decades, and the value of this study lies in the combination of evaluation axes rather than in benchmarking itself (Table 8). Earlier work established much of what is known: comparative behavior across algorithms and imaging modalities [3,4], sensitivity to imaging conditions in shape-from-focus together with the difficulty of defining an absolute ranking [5], behavior in hyperspectral microscopy [6], and quantitative descriptors of focus-curve shape [7]. What is added here is the joint treatment of five smear-microscopy domains at native resolution, a disclosed and perturbed reference construction, ten criteria evaluated as one declared policy including computational cost, and the separation of sampling uncertainty from policy sensitivity, with per-criterion comparisons alongside the aggregate rankings.

### 4.7. Scope, Limitations and Future Work

The evidence concerns five public domains, a frozen candidate pool and explicit scoring choices. REF-B contains self-including and correlated votes; REF-A excludes only one named entry. REF-C tests one alternative, overlapping voter composition and only the localization ordering of the entries outside its voter set. None establishes optical ground truth. WBC can be clustered by slide but not patient, and higher-level grouping is unavailable in the other domains. Runtime describes one environment and selected images, controlled resampling uses 15 stacks, and held-out responses inform GP retention. These conditions define the scope of the reported comparisons.

Three directions follow from these bounds. Anchoring the evaluation to a blinded expert assessment or hardware-calibrated focal intervals would replace consensus agreement with an independent target and is the single most valuable extension. Broadening the library to phase-congruency, Riesz-transform, steerable-filter and shape-from-defocus operators would test whether the gradient tier persists against operator classes not represented here. Finally, the leading gradients should be tested inside a closed-loop controller on prospective acquisitions, where instrument calibration, acceptable-focus thresholds and control performance must be defined in advance. The evaluation protocol provides a reproducible starting structure for each.

## 5. Conclusions

Across 26,100 z-stacks from five smear-microscopy domains, gradient responses formed a strong leading tier under the common REF-B target and primary ten-criterion policy. Variance of Gradient ranked first in all 1000 clustered bootstrap replicates and in 67.9% of sampled criterion-weight configurations. Brenner Gradient provided lower mean consensus deviation and lower measured kernel cost. The equal-domain localization ordering was unchanged under REF-C despite substantial shifts in the reference plane.

A leading gradient response is therefore a practical starting candidate for retrospective focal-plane selection, with the final choice guided by localization tolerance, curve characteristics, computational budget and image sampling. Both criterion priorities and resampling can alter individual rankings; shortlisted operators should be checked under the intended acquisition resolution and preprocessing conditions.

The contribution is an integrated, auditable framework for comparing operator choices under explicit reference, aggregation and scoring conditions. Its localization values describe consensus agreement, while prospective use in a microscope requires independent focus validation and system-level assessment.

## Figures and Tables

**Table 1:** Dataset and acquisition metadata from the source records [20,21,22,23,24]. Dimensions are in pixels and refer to the released images; no additional resizing or cropping was applied in the primary analysis. NA: numerical aperture; n/a: not applicable Unreported fields were not inferred.

Domain	Stacks	Planes/Stack	Dimensions (Pixels)	Axial Step (µm)	Objective/NA	Licence
WBC	25,773	10	200 × 200	0.4	50×/not reported	CC0
TBI	30	10	2112 × 2816	2.5	100× oil/1.25	not reported
PBS	77	9–15	2160 × 2560	0.5	100× oil/1.4	CC0
BMA	38	14	2160 × 2560	0.5	100× oil/1.4	CC0
TBF	182	14	2160 × 2560	0.5	100× oil/1.4	CC0
Total	26,100	9–15	n/a	n/a	n/a	n/a

**Table 2:** Reference constructions and scope. All use plurality voting over per-entry peak indices, upper–middle tie-breaking and endpoint exclusion for stacks with at least three planes. REF-B is the common primary target; REF-A and REF-C answer separate diagnostic questions. None provide independent optical ground truth.

Reference	Voter Set	Comparison Scope	Purpose
REF-B	Ten registered voters; registry in Table A1	Same target for all 32 entries	Primary common target for localization
REF-A	REF-B minus the named entry if it is a voter	Index changes for 10 voters; unchanged for 22 non-voters	Single-exclusion index sensitivity; no full-G reranking
REF-C	4 non-derivative entries from REF-B; no gradient or Laplacian voters	Localization ordering of entries outside its voter set	Family-shift diagnostic; no full-G reranking

**Table 3:** Criterion weights for the primary engineering policy. Alternative deterministic and Dirichlet weightings are evaluated in sensitivity analysis. Preferred direction refers to the raw criterion before alignment.

Criterion	Weight	Role	Preferred Raw Value
Absolute peak-localization error	0.20	Primary localization criterion	Lower
Range around global maximum	0.15	Plateau control near the peak	Lower
False-maxima count	0.10	Anti-multimodality	Lower
Full width at half maximum	0.10	Peak sharpness	Lower
Focus-curve roughness	0.10	Smoothness of the focus curve	Lower
Steep-to-gradual slope ratio	0.10	Focus-transition contrast	Higher
Execution time per plane	0.10	Computational cost	Lower
Steep-slope width	0.05	Transition-region width	Lower
Curvature at peak	0.05	Local peak sharpness	Higher
Focus-curve stability under additive image noise	0.05	Image-noise robustness	Lower

**Table 4:** Leading entries under the primary policy: REF-B, equal-domain aggregation and α = 0.7. G is relative to the frozen pool; lower is better. μ is the equal-domain mean of the five domain scores and σ their population standard deviation. The complete catalogue appears in Table A1.

Rank	Entry	G	μ	σ
1	Variance of Gradient	0.1058	0.1435	0.0181
2	Tenengrad	0.1103	0.1494	0.0190
3	Squared Gradient	0.1105	0.1496	0.0191
4	Energy of Gradient	0.1105	0.1496	0.0192
5	Gradient Squared Energy	0.1105	0.1496	0.0192
6	Brenner Gradient	0.1143	0.1538	0.0222
7	Modified Tenengrad	0.1250	0.1680	0.0247
8	Fourier-Transform Sharpness Index	0.1360	0.1803	0.0324
9	DCT Focus Measure	0.1972	0.2381	0.1020
10	Roberts Focus Measure	0.1989	0.2254	0.1370

**Table 5:** Selected entries compared by aggregate score, consensus localization and computational cost. Mean deviation is the equal-domain mean of domain-specific absolute displacement from REF-B; exact and within-one-plane percentages summarize the corpus. Kernel times are the equal-domain and slice-count-weighted means of the same five domain medians.

Entry	G	Mean Deviation (Planes; Macro)	Exact (%; Corpus)	Within 1 (%; Corpus)	Kernel (ms; Macro)	Kernel (ms; Slice-Weighted)
Variance of Gradient	0.1058	0.146	89.4	99.8	29.1	0.72
Tenengrad	0.1103	0.106	97.7	99.9	21.2	0.53
Brenner Gradient	0.1143	0.072	92.5	99.8	11.1	0.27
Modified Tenengrad	0.1250	0.112	96.5	99.9	288.2	6.83
Fourier-Transform Sharpness Index	0.1360	0.080	92.5	99.8	161.0	3.86
Normalized Variance	0.2202	0.939	1.0	13.0	14.3	0.38

**Table 6:** REF-B/REF-C reference and localization comparisons. Panel A reports reference-index agreement and displacement by domain. Panel B summarizes equal-domain mean absolute-displacement ranking for the entries outside the REF-C voter set under both targets. Rank agreement is specific to this metric and eligible set, not the full ten-criterion score or optical ground truth.

**A. Reference-Index Agreement**						
Domain	Stacks	Exact (%)	Within 1 (%)	Mean shift (planes)	90th percentile (planes)	Maximum (planes)
WBC	25,773	49.4	65.9	0.950	2	7
TBI	30	100.0	100.0	0.000	0	0
PBS	77	71.4	79.2	0.818	3	8
BMA	38	94.7	100.0	0.053	0	1
TBF	182	91.8	100.0	0.082	0	1
**B. Localization-Order Agreement Across the Eligible Entries**						
Entries compared		Spearman ρ	Maximum rank shift	Top five overlap		Top ten overlap
28		1.000	0	5/5		10/10

**Table 7:** Descriptive leave-one-domain-out comparison of the best retained composite and best selected single terminal on each held-out domain. Lower scores are better. Held-out responses informed post-evolution retention, so the scores are not unbiased validation estimates. Differences are reported at the precision of the analysis outputs.

Held-Out Domain	Best Retained Composite Score	Best Selected Terminal Score	Best Selected Single Terminal	Difference (Composite−Single)
WBC	0.1692	0.1161	Fourier high-frequency energy ratio	+0.0531
TBI	0.2688	0.1304	Fourier-transform sharpness index	+0.1384
PBS	0.2957	0.1627	Squared Gradient	+0.1330
BMA	0.2769	0.1452	Squared Gradient	+0.1318
TBF	0.2357	0.1016	Gradient Squared Energy	+0.1341

**Table 8:** Representative focus-measure benchmarks and their evaluation settings. The comparison summarizes methodological emphasis rather than claiming that unlisted analyses were absent. The contribution here is the integration of evaluation dimensions on one five-domain corpus.

Study	Imaging Context	Entries	Focus Assessment	Criteria/Emphasis	Conditions Examined
Groen et al. [3]	Grid, metaphase and portrait images	11	Visually selected in-focus planes	Peak location and widths	Image content; derivative implementation
Sun et al. [4]	Brightfield, phase contrast and DIC microscopy	18	Microscopy autofocus comparison	Accuracy, range, false maxima and cost	Samples, modes, magnification and noise
Pertuz et al. [5]	Shape-from-focus sequences	36 (initial set)	Focus-based depth reconstruction	Depth-estimation performance	Noise, contrast, saturation and window size
Nasibov [6]	Visible and near-infrared hyperspectral microscopy	22	PCA-derived reference focus	Agreement with reference focus	Spectral imaging conditions
Piao et al. [7]	Optical microscopy image sequences	Selected subset	Focus-curve morphology	Slope-region width, slope ratio, curvature and RRMSE	Image content, acquisition parameters and noise
This study	Five smear domains; 26,100 z-stacks	32 entries	REF-B; REF-A index and REF-C localization diagnostics	Ten criteria, including kernel cost	Clustered bootstrap; aggregation, weight and image-sampling sensitivity

## Data Availability

The focus-measure library, consensus-label construction scripts, leave-one-domain-out genetic-programming configuration, multi-criteria scoring pipeline, sensitivity-analysis scripts, exported tables and figure-generation scripts are openly available at https://github.com/DinethHewa/autofocusBenchmark (accessed on 10 August 2026) and archived at Zenodo, doi: 10.5281/zenodo.22261013; Table S23 inventories the machine-readable release and identifies the file supporting each reported analysis. The image datasets analyzed in this study are third-party public resources available from their original sources as cited in references [20,21,22,23,24]; raw images are not redistributed here.

## Supplementary Materials

The following supporting information can be downloaded at: https://www.mdpi.com/article/10.3390/jimaging12090434/s1, Table S1: The 32 catalogue entries with assigned family, representative source, implementation correspondence group and reference membership; Table S2: Implementation correspondence groups established by the all-pairs curve audit over 26,100 stacks; Table S3: Effect of the histogram-binning convention on Histogram Entropy, by domain; Figure S1: Effect of the histogram-binning convention on the thick-blood-film domain, the only 16-bit source among the five; Table S4: Criterion weights under the primary engineering policy, with the internal identifier used in the machine-readable release; Table S5: Sensitivity of the leading entry to the mean-dispersion parameter alpha; Table S6: Sensitivity of the leading entry to deterministic reweighting; Table S7: Reference-index sensitivity to single-voter exclusion (REF-A), by excluded voter and domain; Figure S2: Reference-index stability under single-voter exclusion (REF-A); Table S8: Reference-index agreement between REF-B and REF-C, by domain; Figure S3: Displacement of the reference index between REF-B and REF-C, by domain; Table S9: Equal-domain mean absolute displacement and localization rank under REF-B and REF-C for the 28 entries outside the REF-C voter set; Figure S4: Equal-domain mean absolute displacement under REF-B and REF-C for the 28 entries outside the REF-C voter set, connected in pairs; Table S10: Complete ranking of the 32 catalogue entries under the primary policy; Table S11: Domain scores for the 32 catalogue entries under the primary policy; Table S12: Consensus localization under REF-B for the leading catalogue entries, by domain; Table S13: Raw, directionally unaligned values of the four highest-weighted curve criteria for all 32 entries, expressed as the equal-domain mean of the five domain-specific values; Table S14: Clustered bootstrap results for all 32 entries, from 1000 paired replicates with the white-cell domain clustered by slide; Figure S5: Clustered-bootstrap percentile intervals for G across all 32 catalogue entries, from 1000 paired replicates with the white-cell domain clustered by slide; Table S15: Criterion-weight sensitivity for all 32 entries, from 1000 draws of a flat Dirichlet distribution over the ten weights; Figure S6: Criterion-weight sensitivity from 1000 draws of a flat Dirichlet distribution over the ten weights, for every entry reaching the top five in more than two per cent of draws; Table S16: Mean absolute displacement from REF-B under four aggregation estimands, for all 32 entries; Table S17: Leave-one-domain-out ranks for all 32 entries; Table S18: Median operator-kernel time per plane at native acquisition dimensions, by operator and domain, in milliseconds; Table S19: Equal-domain macro and plane-count-weighted micro kernel times per plane, in milliseconds, at three standardized image sizes and at native acquisition dimensions; Figure S7: Equal-domain median operator-kernel time against square image side, on logarithmic axes; Table S20: Rank within the controlled resampling experiment for all 32 entries under five representative conditions; Figure S8: Rank of every catalogue entry under all 14 condition of the controlled resampling experiment, ordered by rank at native acquisition dimensions; Table S21: Descriptive associations between rank movement and the change in each contributing descriptor, by resampling condition; Table S22: Provenance of the 14 retained symbolic composites; Figure S9: Best retained symbolic composite against the best selected single terminal on each held-out domain; Table S23: Inventory of the machine-readable release, grouped by the analysis it supports.

## Appendix A

**Table A1:** The 32 catalogue entries, assigned families, representative sources and reference membership. B and C denote membership of the REF-B and REF-C voter sets; REF-A excludes the named REF-B voter only. Implementation correspondences among entries are documented in the Supplementary Materials.

Focus Measure	Family	Source	Reference B/C
Variance of Gradient	Gradient	[3]	B
Gradient Squared Energy	Gradient	[5]	–
Squared Gradient	Gradient	[3]	–
Energy of Gradient	Gradient	[5]	B
Tenengrad	Gradient	[26]	B
Brenner Gradient	Gradient	[30]	B
Modified Tenengrad	Gradient	[26]	–
Roberts Focus Measure	Gradient	[31]	–
Spatial Frequency	Gradient	[5]	–
SumModified Laplacian	Laplacian	[32]	B
Variance of Laplacian	Laplacian	[5]	B
Laplacian Energy	Laplacian	[5]	–
Mean Absolute Laplacian	Laplacian	[5]	–
Fourier-Transform Sharpness Index	Frequency	[25]	B, C
DCT Focus Measure	Frequency	[5]	–
Fourier High Frequency Energy Ratio	Frequency	[25]	–
Wavelet W1	Wavelet	[25]	–
Wavelet W2	Wavelet	[25]	–
Wavelet W3	Wavelet	[25]	–
Wavelet Detail Energy (db1)	Wavelet	[25]	–
Curvelet Transform Sharpness Index	Wavelet	[25]	–
Normalized Variance	Statistical	[3]	B, C
Intensity Variance	Statistical	[3]	–
Absolute Central Moment 2	Statistical	[5]	–
Absolute Central Moment 4	Statistical	[5]	–
Intensity Range Index	Statistical	[5]	–
Image Power	Statistical	[5]	–
Mean Intensity	Statistical	[3]	–
Maximal Intensity	Statistical	[3]	–
Intensity Skewness Index	Statistical	[5]	–
GLCM Contrast	Texture	[5]	B, C
Histogram Entropy	Entropy	[3]	B, C

**Table A2:** Operational definitions before directional alignment. Let *f* be a min–max-normalized focus curve with *n* samples, p its selected index, y the reference index and *ε* a numerical stabilizer. Widths and localization deviations use acquired-plane units unless stated otherwise. Higher slope ratio and peak curvature, and lower values of the other criteria, are preferred under the primary policy.

Criterion	Definition
Absolute peak-localization error	Given a reference index y and predicted peak p, the error is |*p − y|.*
Range around global maximum	Width of the contiguous region around the global maximum whose values remain at least 0.95 of the peak value.
False-maxima count	Count of strict local maxima excluding the selected global peak.
Full width at half maximum	Inclusive span from the first to the last sample at or above half of the peak value; it can include below-threshold samples between them. Set to n if the peak is zero.
Focus-curve roughness	Mean squared second difference of the normalized focus curve, mean((Δ*^2^f*)^2^); set to 0 for curves with fewer than three samples. This describes curve smoothness, not sensor noise in the image.
Steep-to-gradual slope ratio	Mean absolute first difference adjacent to the peak divided by (mean absolute background first difference excluding peak-adjacent differences + *ε*).
Execution time per plane	Median operator-kernel wall-clock time per image plane after two warm-ups and seven repeats, with randomized domain–resolution–operator job order. Aggregation is defined in Section 2.7.
Steep-slope width	Distance between the nearest local minima on either side of the peak; a boundary is used where a side has no local minimum.
Curvature at peak	For interior peaks, *max*(0, *−*(*f*[*p −* 1] *−* 2*f*[*p*] + *f*[*p* + 1])); 0 at endpoints.
Focus-curve stability under additive image noise	Relative root-mean-square difference between the clean normalized focus curve f and perturbed curve *f′: sqrt(mean((f − f′)*^2^*))/(sqrt(mean(f*^2^*)) + ε*). The perturbation uses Gaussian noise with standard deviation 0.01 after per-plane min–max normalization, followed by clipping to [0, 1]. It is an idealized perturbation, not a calibrated sensor-noise model.

Noise implementation note: one perturbation realization per stack uses generator seed 123, evaluated on the first min(200, N) stacks of each domain, where N is the domain stack count. The noisy-image preprocessing is stated explicitly because its relationship to the clean path affects interpretation of the criterion.
