# Supplementary material for: Cross-Domain Benchmarking of Focus Measures for Smear-Microscopy Autofocus Under a Consensus-Audited Reference
Source: J Imaging. 2026 Sep 10;12(9):434. doi: 10.3390/jimaging12090434 (PMC13608100; doi:10.3390/jimaging12090434)
Supplement: Supplementary file 1 [file jimaging-12-00434-s001.zip › jimaging-4524210-supplementary.pdf]

Journal of Imaging

MDPI

Supplementary Materials

Cross-Domain Benchmarking of Focus Measures for Smear-Microscopy Autofocus Under a Consensus-Audited Reference

Dineth Hewavitharana, Dumith Jayathilaka *, Palitha Dassanayake and Ranjith Amarasinghe

Department of Mechanical Engineering, Faculty of Engineering, University of Moratuwa, Katubedda 10400, Sri Lanka; hewavitharanadc.24@uom.lk (D.H.); palitha@uom.lk (P.D.); ranama@uom.lk (R.A.)
* Correspondence: dumithj@uom.lk

This document supports the manuscript identified above. It records the frozen focus-measure library and its implementation correspondences, the curve-computation and normalization conventions, the source-encoding and histogram-binning convention, the criterion definitions and weighting policy, the three reference constructions and their diagnostics, the complete ranking of all 32 catalogue entries, raw and normalized criterion values, the clustered-bootstrap and scoring-policy sensitivity analyses, the computational-cost and image-resampling results, the provenance of the exploratory symbolic composites, and an inventory of the machine-readable release.

All values are reproduced from the executed analysis outputs. Planes are reported in acquired-plane units and are zero-based internally; reported displacements are unchanged by index origin. Percentages are computed over the stack counts given in each table. Where a quantity is a decision score rather than a physical measurement this is stated in the corresponding caption.

Deterministic configuration. Candidate pool 32 entries (frozen); primary reference REF-B_fixed_ten_diagnostic; alpha = 0.70; bootstrap replicates 1000; balanced subsampling 30 stacks per domain with 1000 repeats; Dirichlet draws 1000; global seed 4524210. Highest resampling unit: WBC slide (214 official clusters); stack for TBI, PBS, BMA, and TBF.

S1. Focus-Measure Library and Provenance

The candidate pool was frozen before scoring and contains 32 catalogue entries spanning gradient, Laplacian, statistical, entropy, texture, frequency and wavelet-labelled families. Entries are retained under their published names so that the pool matches the operator lists from which they were drawn.

A subset of entries share a common underlying implementation and therefore return the same focus response. The correspondence was established empirically rather than inferred from the operator definitions: every pair of the 32 entries was compared on the nine curve-derived criteria across all five domains and all 26,100 stacks. Eleven pairs agree exactly, forming four correspondence groups, so the 32 catalogue entries yield 25 distinct focus responses. Within each group the nine curve-derived criteria are identical and only the measured operator-kernel time differs, which is why members of a group occupy adjacent ranks separated by cost alone. Ranks are interpreted with this in mind throughout the manuscript.

Table S1. The 32 catalogue entries with assigned family, representative source, implementation correspondence group and reference membership. Source numbers refer to the reference list of the main manuscript. Correspondence groups are defined in Table S2; a blank entry indicates a response shared with no other catalogue entry. B and C denote membership of the REF-B and REF-C voter sets; REF-A excludes the named REF-B voter only.

Focus Measure	Family	Source	Group	REF-B / REF-C
Variance of Gradient	Gradient	[3]	–	B
Gradient Squared Energy	Gradient	[5]	E1	–
Squared Gradient	Gradient	[3]	E1	–
Energy of Gradient	Gradient	[5]	E1	B
Tenengrad	Gradient	[26]	E1	B
Brenner Gradient	Gradient	[30]	–	B
Modified Tenengrad	Gradient	[26]	–	–
Roberts Focus Measure	Gradient	[31]	–	–
Spatial Frequency	Gradient	[5]	–	–
Sum Modified Laplacian	Laplacian	[32]	–	B
Variance of Laplacian	Laplacian	[5]	–	B
Laplacian Energy	Laplacian	[5]	–	–
Mean Absolute Laplacian	Laplacian	[5]	–	–
Fourier Transform Sharpness Index	Frequency	[25]	–	B, C
DCT Focus Measure	Frequency	[5]	–	–
Fourier High Frequency Energy Ratio	Frequency	[25]	–	–
Wavelet W1	Wavelet	[25]	E2	–
Wavelet W2	Wavelet	[25]	E2	–
Wavelet W3	Wavelet	[25]	E2	–
Wavelet Detail Energy (db1)	Wavelet	[25]	E3	–
Curvelet Transform Sharpness Index	Wavelet	[25]	E3	–
Normalized Variance	Statistical	[3]	–	B, C
Intensity Variance	Statistical	[3]	E4	–
Absolute Central Moment 2	Statistical	[5]	E4	–
Absolute Central Moment 4	Statistical	[5]	–	–
Intensity Range Index	Statistical	[5]	–	–
Image Power	Statistical	[5]	–	–
Mean Intensity	Statistical	[3]	–	–
Maximal Intensity	Statistical	[3]	–	–
Intensity Skewness Index	Statistical	[5]	–	–
GLCM Contrast	Texture	[5]	–	B, C
Histogram Entropy	Entropy	[3]	–	B, C

Table S2. Implementation correspondence groups established by the all-pairs curve audit over 26,100 stacks. Identical pairs is the number of exactly agreeing pairs contributed by each group. Within every group the nine curve-derived criteria coincide and only measured operator-kernel time differs.

Group	Member Entries	Basis of the Correspondence	Identical Pairs
E1	Tenengrad; Squared Gradient; Energy of Gradient; Gradient Squared Energy	Common Sobel gradient-energy implementation	6
E2	Wavelet W1; Wavelet W2; Wavelet W3	The implementation selects the finest detail level irrespective of the requested decomposition depth	3
E3	Wavelet Detail Energy (db1); Curvelet Transform Sharpness Index	The curvelet-labelled entry delegates to the db1 detail-energy routine	1

https://doi.org/10.3390/jimaging12090434

E4	Intensity Variance; Absolute Central Moment 2	The second absolute central moment is algebraically the variance	1

Totals: four groups, 11 identical pairs, 25 distinct responses from 32 catalogue entries.

S2. Focus-Curve Computation and Normalization Conventions

Each operator returns one scalar per acquired plane. The source encoding range is identified before floating-point conversion and mapped consistently to the unit interval. Within a stack the focus curve is min-max normalized to the unit interval before the curve-shape criteria are computed; constant curves are mapped to zero.

The predicted focal index of an evaluated candidate is the global maximum over the whole curve, including the two endpoint planes, with ties resolved by the upper central index among the sorted tied indices. This differs deliberately from the voter rule used to build a reference, which excludes the two endpoint planes whenever a stack contains at least three planes. The consequence is an asymmetry: an evaluated candidate may select a plane that no reference can occupy. Across all 32 entries and 26,100 stacks, 9.0% of predictions fall on an endpoint, ranging from 4.7% in BMA to 35.5% in TBF, and 0.20% to 0.34% for the six leading catalogue entries.

Source filenames are ordered by natural numeric sort rather than lexicographic sort, so that plane 10 follows plane 9. Internal indices are zero-based. Reported displacements are differences between indices and are therefore unchanged by index origin.

S3. Source Encoding Range and Histogram Binning

Histogram Entropy is binned into 256 fixed bins over the source encoding range identified before floating-point conversion. Binning on the stored range rather than on post-conversion values is necessary because a 16-bit source would otherwise contribute only its lowest 256 levels, which drives the computed entropy to a constant across the stack and destroys the focus response.

The submitted implementation applied a fixed 0 to 256 range after conversion. Table S3 reports the effect of the corrected convention on each domain. Only the thick-blood-film domain is affected, because it is the only 16-bit source among the five: all 182 of its curves were constant under the submitted convention and none is constant under the corrected convention, every stack changes its entropy peak, and the largest absolute change in a computed value is 5.126 bits. The four 8-bit domains are unchanged to the precision of the computation.

This correction is consequential beyond the entropy criterion itself, because Histogram Entropy is a registered REF-B and REF-C voter as well as a scored candidate. The reference indices and the ranking were regenerated from the corrected curves. Eleven dtype and regression tests enforce the convention, covering 8-bit and 16-bit inputs, constant images, and the boundary between the stored range and the normalized range.

Table S3. Effect of the histogram-binning convention on Histogram Entropy, by domain. Constant curves are stacks whose computed entropy does not vary across planes and which therefore carry no focus information. Peak changes counts stacks whose selected entropy maximum moves between the submitted and corrected conventions. The maximum absolute change is expressed in bits of computed entropy.

Domain	Stacks	Planes	Constant Curves, Submitted	Constant Curves, Corrected	Stacks with a Changed Peak	Maximum Absolute Change (bits)
WBC	25,773	257,730	0	0	0	0.000
TBI	30	300	0	0	0	0.000
PBS	77	1035	0	0	0	0.000

BMA	38	532	0	0	0	0.000
TBF	182	2548	182	0	182	5.126

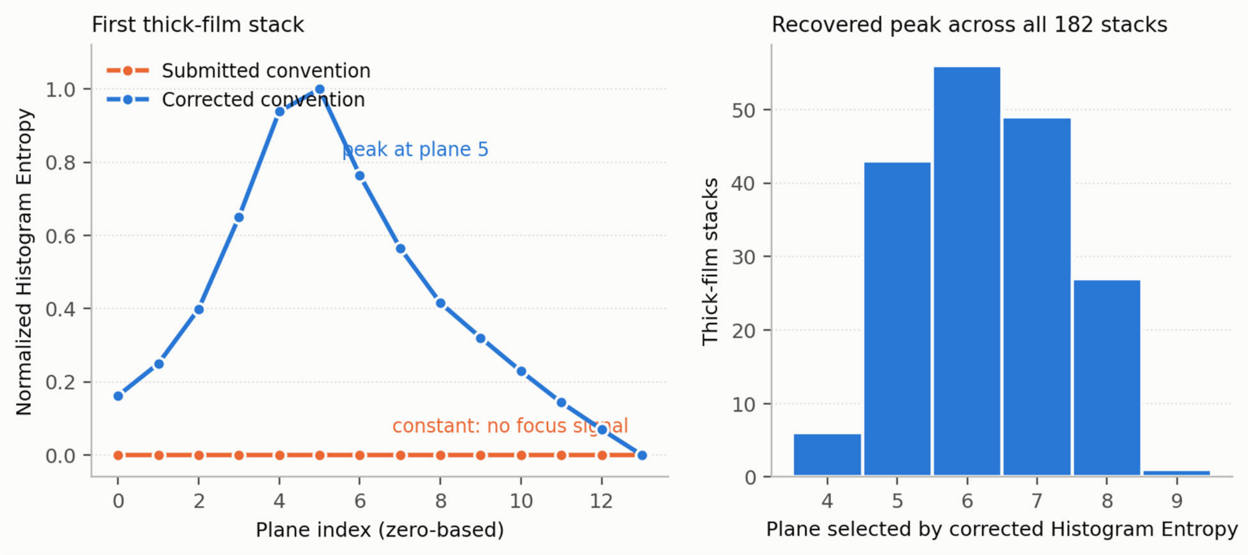

Figure S1. Effect of the histogram-binning convention on the thick-blood-film domain, the only 16-bit source among the five. Left: the first stack. Under the submitted convention the computed entropy is constant across all planes, so the curve carries no focus information and its normalized form is identically zero; under the corrected convention the same stack produces a well-formed unimodal response. Right: the plane selected by the corrected Histogram Entropy across all 182 stacks of the domain, showing that the recovered signal varies between stacks rather than collapsing to a fixed plane. The four 8-bit domains are unchanged by the convention, as reported in Table S3.

S4. Evaluation Criteria, Weighting Policy and Scoring Sensitivity

Ten criteria are combined into the cross-domain decision score G. Raw criterion values are directionally aligned so that lower is better, then normalized across the frozen 32-entry pool within each domain-criterion cell. G is therefore a candidate-pool-relative decision score and not an absolute performance scale: adding or removing candidates can change it. Raw values are released alongside the normalized values for this reason and are summarized in Section S7.

Both the criterion weights and the mean-dispersion trade-off parameter alpha are author-defined engineering choices. They were not elicited from domain experts and were not optimized against the data, and they are not claimed to be optimal or to have been prospectively prespecified. Tables S5 and S6 report how far the leading result depends on them.

Table S4. Criterion weights under the primary engineering policy, with the internal identifier used in the machine-readable release. Preferred direction refers to the raw criterion before directional alignment. Weights sum to one.

Criterion	Identifier in the Release	Weight	Preferred Raw Value
Absolute peak-localization error	absolute_peak_localization_error	0.20	Lower
Range around global maximum	range_around_global_maximum	0.15	Lower
False-maxima count	false_maxima_count	0.10	Lower
Full width at half maximum	fwhm	0.10	Lower
Focus-curve roughness	noise_level	0.10	Lower
Steep-to-gradual slope ratio	steep_to_gradual_slope_ratio	0.10	Higher
Execution time per plane	execution_time_per_slice	0.10	Lower

https://doi.org/10.3390/jimaging12090434

Steep-slope width	steep_slope_width	0.05	Lower
Curvature at peak	curvature_at_peak	0.05	Higher
Focus-curve stability under additive image noise	rrmse_under_additive_noise	0.05	Lower

Table S5. Sensitivity of the leading entry to the mean-dispersion parameter alpha. G combines the equal-domain mean and the population standard deviation of the five domain scores as alpha times the mean plus one minus alpha times the dispersion. The rank-one entry is unchanged across the full range; the score changes because the two components are weighted differently, not because performance changes. This demonstrates insensitivity over the tested range and does not establish an optimal alpha.

Alpha	Rank-one Entry	G of the Rank-one Entry
0.0	Variance of Gradient	0.0181
0.1	Variance of Gradient	0.0306
0.2	Variance of Gradient	0.0432
0.3	Variance of Gradient	0.0557
0.4	Variance of Gradient	0.0682
0.5	Variance of Gradient	0.0808
0.6	Variance of Gradient	0.0933
0.7	Variance of Gradient	0.1058
0.8	Variance of Gradient	0.1184
0.9	Variance of Gradient	0.1309
1.0	Variance of Gradient	0.1435

Table S6. Sensitivity of the leading entry to deterministic reweighting. Each scheme redistributes the criterion weights and the score is recomputed over the same frozen pool. Scores are not comparable between schemes because each scheme defines a different quantity.

Weighting Scheme	Rank-one Entry	G of the Rank-one Entry
submitted	Variance of Gradient	0.1058
equal	Variance of Gradient	0.1467
localization heavy	Variance of Gradient	0.0432
runtime 0.10	Variance of Gradient	0.1058
runtime 0.05	Variance of Gradient	0.1107
runtime 0	Variance of Gradient	0.1160

S5. Reference Constructions and Their Diagnostics

No independent hardware-calibrated or expert-labelled focal index is available across all five resources, so the reference is algorithmic. It is a plurality vote over the per-voter selected peak indices, not an arithmetic mean of normalized voter curves. Votes are counted at each plane and the selected plane is the one receiving the most votes, which need not be more than half of them. Ties are resolved by sorting the tied indices and returning the middle element, taking the upper-middle element when the number of tied indices is even; the same rule resolves ties inside an individual voter curve. Voter eligibility excludes the two endpoint planes whenever a stack contains at least three planes. One reference is constructed per stack and per preprocessing condition.

REF-B is the primary target. Its registry contains Tenengrad, Brenner Gradient, Energy of Gradient, Variance of Gradient, Variance of Laplacian, Sum Modified Laplacian, Normalized Variance, Histogram Entropy, GLCM Contrast and the Fourier Transform Sharpness Index, and it is applied identically to all 32 candidates. REF-B includes direct self-voting and correlated families and is a common diagnostic target, not an independent optical standard.

https://doi.org/10.3390/jimaging12090434

REF-A excludes the evaluated entry from the registry when that entry is a registered voter, and otherwise retains the full registry. It quantifies how far the reference index moves when one named voter is removed. It is not an independent target and it is not a full-score reranking: removing an entry's direct vote does not remove a related or identical response that remains in the registry.

REF-C restricts the registry to Normalized Variance, Histogram Entropy, GLCM Contrast and the Fourier Transform Sharpness Index, a subset of REF-B containing no gradient or Laplacian entry. It is a family-shift diagnostic and is not assumed to be more accurate than REF-B: disagreement between the two measures sensitivity to voter composition, not the error of either against optical best focus. Its localization comparison covers the entries outside its voter set.

Table S7. Reference-index sensitivity to single-voter exclusion (REF-A), by excluded voter and domain. Each cell gives the percentage of stacks whose plurality outcome is unchanged when that voter is removed from the registry, and, in parentheses, the mean absolute displacement of the reference index in planes. The reference index is invariant to single-voter removal on TBI and BMA. These figures characterize the reference index alone and are not evidence that operator rankings are unchanged.

Excluded Voter	WBC	TBI	PBS	BMA	TBF
Tenengrad	96.0 (0.041)	100.0 (0.000)	97.4 (0.117)	100.0 (0.000)	96.2 (0.170)
Brenner Gradient	97.2 (0.029)	100.0 (0.000)	96.1 (0.156)	100.0 (0.000)	96.2 (0.209)
Energy of Gradient	96.0 (0.041)	100.0 (0.000)	97.4 (0.117)	100.0 (0.000)	96.2 (0.170)
Variance of Gradient	98.7 (0.013)	100.0 (0.000)	97.4 (0.117)	100.0 (0.000)	96.7 (0.104)
Variance of Laplacian	99.3 (0.007)	100.0 (0.000)	94.8 (0.156)	100.0 (0.000)	100.0 (0.000)
Sum Modified Laplacian	99.3 (0.007)	100.0 (0.000)	94.8 (0.156)	100.0 (0.000)	100.0 (0.000)
Normalized Variance	99.9 (0.001)	100.0 (0.000)	98.7 (0.078)	100.0 (0.000)	98.9 (0.049)
Histogram Entropy	99.7 (0.003)	100.0 (0.000)	94.8 (0.156)	100.0 (0.000)	100.0 (0.000)
GLCM Contrast	98.5 (0.015)	100.0 (0.000)	94.8 (0.156)	100.0 (0.000)	100.0 (0.000)
Fourier Transform Sharpness Index	96.2 (0.039)	100.0 (0.000)	97.4 (0.117)	100.0 (0.000)	95.6 (0.181)

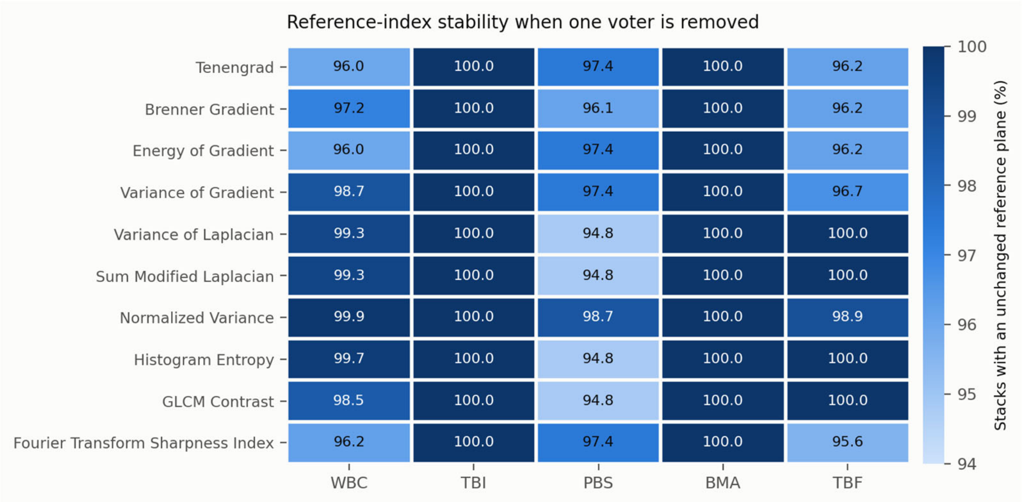

Figure S2. Reference-index stability under single-voter exclusion (REF-A). Each cell is the percentage of stacks whose plurality outcome is unchanged when that voter is removed from the REF-B registry. The reference index is invariant on the sputum and bone-marrow domains for every voter, and the largest effect anywhere leaves 94.8 per cent of stacks unchanged, on the peripheral-blood domain, where four non-gradient voters share that minimum. Mean absolute displacements for the same cells are given in Table S7. These values characterize the reference index alone and are not evidence that operator rankings are unchanged.

Table S8. Reference-index agreement between REF-B and REF-C, by domain. Exact is the percentage of stacks selecting the same plane under both constructions and within one the percentage selecting the same or an adjacent plane. Displacements are in acquired planes. Changing the voter composition moves the reference substantially on the dominant domain.

Domain	Stacks	Exact (%)	Within 1 (%)	Mean	Median	90th Percentile	Maximum
WBC	25,773	49.4	65.9	0.950	1.0	2.0	7
TBI	30	100.0	100.0	0.000	0.0	0.0	0
PBS	77	71.4	79.2	0.818	0.0	3.0	8
BMA	38	94.7	100.0	0.053	0.0	0.0	1
TBF	182	91.8	100.0	0.082	0.0	0.0	1

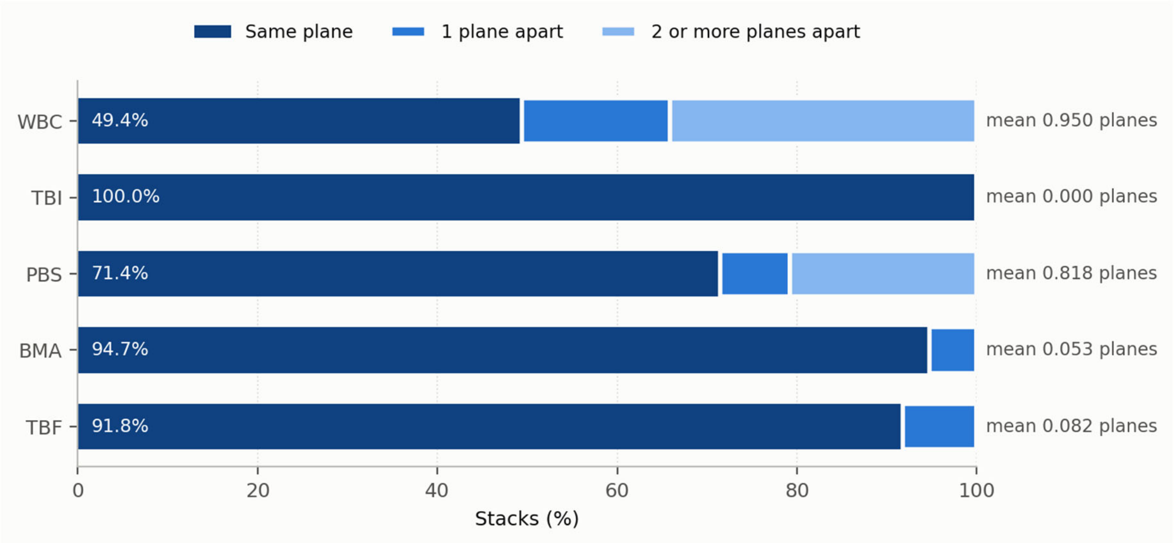

Figure S3. Displacement of the reference index between REF-B and REF-C, by domain. Bars give the percentage of stacks selecting the same plane, an adjacent plane, or a plane two or more away. Removing the gradient and Laplacian voters leaves the reference unchanged on the sputum domain and almost unchanged on bone marrow and thick film, but moves it on roughly half of the white-cell stacks. Absolute localization values are therefore specific to the target used.

Table S9. Equal-domain mean absolute displacement and localization rank under REF-B and REF-C for the 28 entries outside the REF-C voter set. Displacements are in acquired planes and are the equal-domain mean of the five domain-specific values. Ranks are average ranks, so tied entries share a fractional rank. Every entry retains its rank, giving Spearman rho = 1.000 with a maximum rank shift of zero, while absolute displacement inflates under REF-C. Ordering is therefore far more robust to voter composition than magnitude.

Entry	REF-B Displacement	REF-C Displacement	REF-B Rank	REF-C Rank	Rank Shift
Brenner Gradient	0.072	0.412	1	1	0
Energy of Gradient	0.106	0.471	3.5	3.5	0
Gradient Squared Energy	0.106	0.471	3.5	3.5	0
Squared Gradient	0.106	0.471	3.5	3.5	0
Tenengrad	0.106	0.471	3.5	3.5	0
Modified Tenengrad	0.112	0.479	6	6	0
Variance of Gradient	0.146	0.511	7	7	0
Absolute Central Moment 2	0.827	0.542	8.5	8.5	0
Intensity Variance	0.827	0.542	8.5	8.5	0
Absolute Central Moment 4	0.881	0.669	10	10	0

DCT Focus Measure	0.893	1.076	11	11	0
Roberts Focus Measure	0.914	1.177	12	12	0
Spatial Frequency	1.122	1.385	13	13	0
Intensity Range Index	1.236	1.487	14	14	0
Maximal Intensity	1.452	1.579	15	15	0
Fourier High Frequency Energy Ratio	1.615	1.837	16	16	0
Curvelet Transform Sharpness Index	1.643	1.849	19	19	0
Wavelet Detail Energy (db1)	1.643	1.849	19	19	0
Wavelet W1	1.643	1.849	19	19	0
Wavelet W2	1.643	1.849	19	19	0
Wavelet W3	1.643	1.849	19	19	0
Image Power	2.216	2.245	22	22	0
Sum Modified Laplacian	2.572	2.652	23	23	0
Laplacian Energy	2.810	2.890	24.5	24.5	0
Variance of Laplacian	2.810	2.890	24.5	24.5	0
Mean Absolute Laplacian	3.105	3.185	26	26	0
Mean Intensity	4.468	4.775	27	27	0
Intensity Skewness Index	5.596	5.392	28	28	0

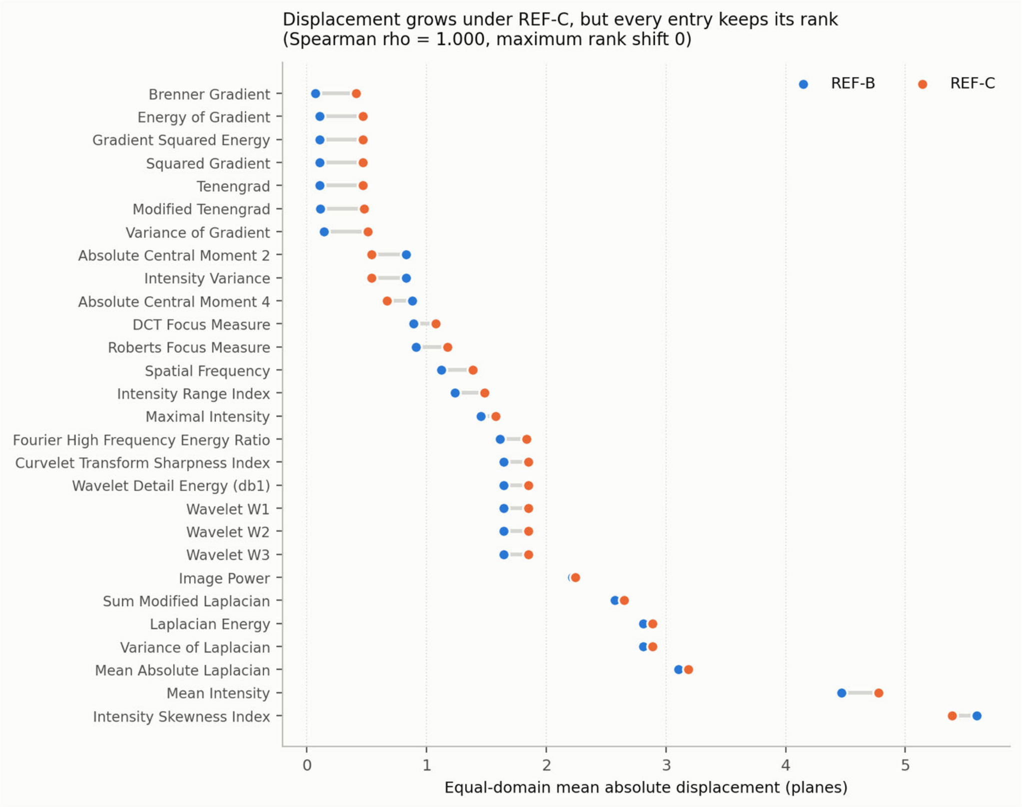

Figure S4. Equal-domain mean absolute displacement under REF-B and REF-C for the 28 entries outside the REF-C voter set, connected in pairs. Displacement grows for most entries under REF-C and falls for a few, yet the vertical ordering is identical under both targets: every entry retains its rank, giving Spearman rho = 1.000 with a maximum rank shift of zero. Ordering is therefore far more robust to voter composition than magnitude. This is empirical rank robustness on this metric and these two operator-derived targets, not a mathematical cancellation property.

S6. Complete Ranking under the Primary Policy

Tables S10 and S11 give the complete ranking of all 32 catalogue entries under REF-B, equal-domain aggregation and alpha = 0.70, together with the five domain scores from

which the aggregate is formed. Lower values are preferred throughout. Entries belonging to one correspondence group occupy adjacent ranks and are separated only by measured operator-kernel time.

Table S10. Complete ranking of the 32 catalogue entries under the primary policy. G is the combined score, mu the equal-domain mean of the five domain scores and sigma their population standard deviation. G is relative to the frozen 32-entry pool and is not an absolute performance scale.

Rank	Entry	G	Mu	Sigma
1	Variance of Gradient	0.1058	0.1435	0.0181
2	Tenengrad	0.1103	0.1494	0.0190
3	Squared Gradient	0.1105	0.1496	0.0191
4	Energy of Gradient	0.1105	0.1496	0.0192
5	Gradient Squared Energy	0.1105	0.1496	0.0192
6	Brenner Gradient	0.1143	0.1538	0.0222
7	Modified Tenengrad	0.1250	0.1680	0.0247
8	Fourier Transform Sharpness Index	0.1360	0.1803	0.0324
9	DCT Focus Measure	0.1972	0.2381	0.1020
10	Roberts Focus Measure	0.1989	0.2254	0.1370
11	Spatial Frequency	0.2040	0.2375	0.1260
12	Wavelet W1	0.2168	0.2585	0.1196
13	Curvelet Transform Sharpness Index	0.2170	0.2587	0.1196
14	Wavelet Detail Energy (db1)	0.2170	0.2588	0.1196
15	Wavelet W3	0.2172	0.2590	0.1198
16	Wavelet W2	0.2177	0.2596	0.1197
17	Absolute Central Moment 4	0.2194	0.2798	0.0784
18	Normalized Variance	0.2202	0.2825	0.0748
19	Intensity Variance	0.2240	0.2864	0.0785
20	Absolute Central Moment 2	0.2244	0.2869	0.0784
21	Fourier High Frequency Energy Ratio	0.2341	0.2772	0.1338
22	Intensity Range Index	0.2397	0.3193	0.0537
23	Histogram Entropy	0.2994	0.3593	0.1597
24	Variance of Laplacian	0.3002	0.3636	0.1522
25	Sum Modified Laplacian	0.3029	0.3675	0.1523
26	Laplacian Energy	0.3069	0.3721	0.1547
27	Image Power	0.3073	0.3949	0.1028
28	Mean Absolute Laplacian	0.3111	0.3837	0.1418
29	Mean Intensity	0.3260	0.4341	0.0738
30	Intensity Skewness Index	0.3461	0.4436	0.1188
31	Maximal Intensity	0.3467	0.4415	0.1256
32	GLCM Contrast	0.3726	0.4449	0.2038

Table S11. Domain scores for the 32 catalogue entries under the primary policy. Each value is the weighted combination of the ten normalized criteria within that domain, before equal-domain aggregation. Lower is better.

Entry	S(WBC)	S(TBI)	S(PBS)	S(BMA)	S(TBF)
Variance of Gradient	0.1222	0.1768	0.1355	0.1421	0.1406
Tenengrad	0.1213	0.1755	0.1650	0.1423	0.1431
Squared Gradient	0.1212	0.1755	0.1656	0.1422	0.1433
Energy of Gradient	0.1212	0.1755	0.1658	0.1420	0.1433
Gradient Squared Energy	0.1212	0.1755	0.1660	0.1422	0.1432
Brenner Gradient	0.1211	0.1855	0.1682	0.1403	0.1537

https://doi.org/10.3390/jimaging12090434

Modified Tenengrad	0.1282	0.2044	0.1783	0.1651	0.1638
Fourier Transform Sharpness Index	0.1487	0.1606	0.2419	0.1791	0.1713
DCT Focus Measure	0.1770	0.1504	0.2451	0.1854	0.4323
Roberts Focus Measure	0.1179	0.1951	0.1771	0.1429	0.4941
Spatial Frequency	0.1372	0.2083	0.2090	0.1502	0.4826
Wavelet W1	0.1536	0.2253	0.2750	0.1584	0.4802
Curvelet Transform Sharpness Index	0.1537	0.2254	0.2754	0.1588	0.4804
Wavelet Detail Energy (db1)	0.1536	0.2255	0.2757	0.1588	0.4802
Wavelet W3	0.1546	0.2232	0.2763	0.1595	0.4812
Wavelet W2	0.1545	0.2262	0.2767	0.1594	0.4814
Absolute Central Moment 4	0.2823	0.1460	0.3545	0.3611	0.2552
Normalized Variance	0.3182	0.1394	0.3367	0.3398	0.2782
Intensity Variance	0.3181	0.1413	0.3596	0.3425	0.2707
Absolute Central Moment 2	0.3182	0.1419	0.3602	0.3429	0.2714
Fourier High Frequency Energy Ratio	0.1176	0.3246	0.2584	0.1801	0.5051
Intensity Range Index	0.3096	0.3493	0.3719	0.3463	0.2196
Histogram Entropy	0.3326	0.1986	0.6059	0.4680	0.1912
Variance of Laplacian	0.1176	0.4863	0.4789	0.2515	0.4835
Sum Modified Laplacian	0.1215	0.4848	0.4602	0.2582	0.5129
Laplacian Energy	0.1201	0.4964	0.4890	0.2617	0.4935
Image Power	0.2769	0.5684	0.3701	0.3173	0.4420
Mean Absolute Laplacian	0.1613	0.5447	0.4760	0.2783	0.4582
Mean Intensity	0.3345	0.5578	0.4162	0.4018	0.4600
Intensity Skewness Index	0.3410	0.6766	0.4078	0.3994	0.3931
Maximal Intensity	0.6180	0.4338	0.4179	0.5042	0.2337
GLCM Contrast	0.2263	0.6596	0.3221	0.2954	0.7213

S7. Raw and Normalized Criterion Values

The complete raw and normalized criterion values for all 32 entries across the five domains and ten criteria are released in machine-readable form, in both long and wide layouts. Tables S12 and S13 reproduce the parts most often needed to check a comparison by hand.

Table S12 gives consensus localization for the leading entries in both acquired planes and micrometres, using each domain's documented axial step. The fractional values are averages of integer plane selections and do not imply sub-plane estimation. The peripheral-blood-smear domain is the hardest for every leading entry, and its within-one-plane rate equals its exact rate, so the residual errors there are displacements of two planes or more rather than near misses.

Table S12. Consensus localization under REF-B for the leading catalogue entries, by domain. Mean displacement is given in acquired planes and converted to micrometres using the documented axial step of each domain, which is 0.4 micrometres for WBC, 2.5 for TBI and 0.5 for PBS, BMA and TBF. Exact is the percentage of stacks selecting the reference plane and within one the percentage selecting the reference or an adjacent plane. These values describe agreement with a declared consensus, not accuracy against an optical best-focus plane.

Entry	Domain	Mean Displacement (Planes)	Mean Displacement (um)	Exact (%)	Within 1 (%)
Variance of Gradient	WBC	0.111	0.044	89.3	99.8
	TBI	0.000	0.000	100.0	100.0
	PBS	0.519	0.260	88.3	88.3

	BMA	0.053	0.026	94.7	100.0
	TBF	0.049	0.025	95.1	100.0
Tenengrad	WBC	0.024	0.010	97.7	99.9
	TBI	0.000	0.000	100.0	100.0
	PBS	0.468	0.234	89.6	89.6
	BMA	0.026	0.013	97.4	100.0
	TBF	0.011	0.005	98.9	100.0
Squared Gradient	WBC	0.024	0.010	97.7	99.9
	TBI	0.000	0.000	100.0	100.0
	PBS	0.468	0.234	89.6	89.6
	BMA	0.026	0.013	97.4	100.0
	TBF	0.011	0.005	98.9	100.0
Energy of Gradient	WBC	0.024	0.010	97.7	99.9
	TBI	0.000	0.000	100.0	100.0
	PBS	0.468	0.234	89.6	89.6
	BMA	0.026	0.013	97.4	100.0
	TBF	0.011	0.005	98.9	100.0
Gradient Squared Energy	WBC	0.024	0.010	97.7	99.9
	TBI	0.000	0.000	100.0	100.0
	PBS	0.468	0.234	89.6	89.6
	BMA	0.026	0.013	97.4	100.0
	TBF	0.011	0.005	98.9	100.0
Brenner Gradient	WBC	0.080	0.032	92.4	99.8
	TBI	0.000	0.000	100.0	100.0
	PBS	0.234	0.117	92.2	97.4
	BMA	0.026	0.013	97.4	100.0
	TBF	0.022	0.011	97.8	100.0
Modified Tenengrad	WBC	0.037	0.015	96.5	99.9
	TBI	0.000	0.000	100.0	100.0
	PBS	0.494	0.247	89.6	89.6
	BMA	0.026	0.013	97.4	100.0
	TBF	0.005	0.003	99.5	100.0
Fourier Transform Sharpness Index	WBC	0.079	0.032	92.4	99.8
	TBI	0.000	0.000	100.0	100.0
	PBS	0.299	0.149	87.0	93.5
	BMA	0.000	0.000	100.0	100.0
	TBF	0.022	0.011	97.8	100.0

Table S13. Raw, directionally unaligned values of the four highest-weighted curve criteria for all 32 entries, expressed as the equal-domain mean of the five domain-specific values. These are the measured quantities before alignment and before pool-relative normalization, and they can therefore be compared without reference to the frozen candidate pool. Localization error, near-peak range, full width at half maximum and false-maxima count are all in acquired-plane units except the count, which is dimensionless. Lower is preferred for all four.

Entry	Peak-Localization Error	Range Around Maximum	False-Maxima Count	FWHM
Variance of Gradient	0.146	1.230	0.181	3.135
Tenengrad	0.106	1.223	0.232	3.317
Squared Gradient	0.106	1.223	0.232	3.317
Energy of Gradient	0.106	1.223	0.232	3.317
Gradient Squared Energy	0.106	1.223	0.232	3.317
Brenner Gradient	0.072	1.224	0.290	3.322

Modified Tenengrad	0.112	1.217	0.232	3.323
Fourier Transform Sharpness Index	0.080	1.332	0.192	4.147
DCT Focus Measure	0.893	1.428	0.472	5.656
Roberts Focus Measure	0.914	1.355	0.605	4.787
Spatial Frequency	1.122	1.350	0.695	5.101
Wavelet W1	1.643	1.256	0.808	4.726
Curvelet Transform Sharpness Index	1.643	1.256	0.808	4.726
Wavelet Detail Energy (db1)	1.643	1.256	0.808	4.726
Wavelet W3	1.643	1.256	0.808	4.726
Wavelet W2	1.643	1.256	0.808	4.726
Absolute Central Moment 4	0.881	1.620	0.376	5.776
Normalized Variance	0.939	1.698	0.273	6.082
Intensity Variance	0.827	1.681	0.378	6.139
Absolute Central Moment 2	0.827	1.681	0.378	6.139
Fourier High Frequency Energy Ratio	1.615	1.206	0.947	6.006
Intensity Range Index	1.236	1.311	1.083	5.349
Histogram Entropy	2.966	1.516	0.431	7.579
Variance of Laplacian	2.810	1.303	1.298	6.567
Sum Modified Laplacian	2.572	1.345	1.252	6.694
Laplacian Energy	2.810	1.303	1.298	6.567
Image Power	2.216	1.641	1.035	8.001
Mean Absolute Laplacian	3.105	1.292	1.341	6.371
Mean Intensity	4.468	1.479	0.992	7.479
Intensity Skewness Index	5.596	1.518	0.198	9.496
Maximal Intensity	1.452	2.324	1.155	6.184
GLCM Contrast	2.330	1.305	1.111	7.311

S8. Sampling Uncertainty and Scoring-Policy Sensitivity

Sampling variability and sensitivity to the scoring policy are different quantities and are reported separately. The ten criteria are related properties of the same focus curves rather than independent experimental replicates, so no inference treats the 50 domain-by-criterion cells as independent blocks.

Sampling uncertainty is estimated by 1000 paired clustered bootstrap replicates. The same sampled units are used across entries, and the criteria, domain scores, G and ranks are recomputed inside each replicate. The white-cell domain is clustered by its 214 official slides, recovered from the released image-to-slide map and reconciled to all 25,773 cached stacks. The remaining four domains are resampled by stack because no higher-level grouping identifier is released. The release does not map the 214 slides to the 72 patients of the source cohort, so residual dependence among slides from one patient cannot be modelled and the intervals may remain optimistic.

Scoring-policy sensitivity is estimated by 1000 draws from a flat Dirichlet distribution over the ten weights. These winner frequencies describe how the recommendation responds to a change of scoring priorities. They are not sampling confidence probabilities and must not be read as such.

Table S14. Clustered bootstrap results for all 32 entries, from 1000 paired replicates with the white-cell domain clustered by slide. The interval is the 95 per cent percentile interval of G. Probabilities are the fraction of replicates in which the entry attains that position. These quantify resampling stability under a fixed scoring policy.

Entry	Mean G	95% Interval	P(Rank 1)	P(Top 3)	P(Top 5)	Median Rank
Variance of Gradient	0.1059	0.1051 to 0.1071	1.000	1.000	1.000	1

https://doi.org/10.3390/jimaging12090434

Tenengrad	0.1104	0.1092 to 0.1122	0.000	1.000	1.000	2
Squared Gradient	0.1106	0.1094 to 0.1124	0.000	0.575	1.000	3
Energy of Gradient	0.1106	0.1094 to 0.1124	0.000	0.424	1.000	4
Gradient Squared Energy	0.1106	0.1094 to 0.1124	0.000	0.000	0.999	5
Brenner Gradient	0.1144	0.1142 to 0.1150	0.000	0.001	0.001	6
Modified Tenengrad	0.1251	0.1240 to 0.1267	0.000	0.000	0.000	7
Fourier Transform Sharpness Index	0.1361	0.1355 to 0.1373	0.000	0.000	0.000	8
DCT Focus Measure	0.1972	0.1934 to 0.2010	0.000	0.000	0.000	9
Roberts Focus Measure	0.1988	0.1952 to 0.2026	0.000	0.000	0.000	10
Spatial Frequency	0.2039	0.2006 to 0.2072	0.000	0.000	0.000	11
Wavelet W1	0.2166	0.2149 to 0.2185	0.000	0.000	0.000	12
Curvelet Transform Sharpness Index	0.2168	0.2150 to 0.2187	0.000	0.000	0.000	13
Wavelet Detail Energy (db1)	0.2168	0.2150 to 0.2187	0.000	0.000	0.000	14
Wavelet W3	0.2170	0.2152 to 0.2189	0.000	0.000	0.000	15
Wavelet W2	0.2174	0.2157 to 0.2194	0.000	0.000	0.000	16
Absolute Central Moment 4	0.2195	0.2168 to 0.2226	0.000	0.000	0.000	17
Normalized Variance	0.2203	0.2182 to 0.2225	0.000	0.000	0.000	18
Intensity Variance	0.2242	0.2224 to 0.2261	0.000	0.000	0.000	19
Absolute Central Moment 2	0.2245	0.2227 to 0.2265	0.000	0.000	0.000	20
Fourier High Frequency Energy Ratio	0.2338	0.2289 to 0.2387	0.000	0.000	0.000	21
Intensity Range Index	0.2393	0.2338 to 0.2462	0.000	0.000	0.000	22
Variance of Laplacian	0.2992	0.2918 to 0.3071	0.000	0.000	0.000	24
Histogram Entropy	0.2994	0.2936 to 0.3048	0.000	0.000	0.000	24
Sum Modified Laplacian	0.3020	0.2942 to 0.3101	0.000	0.000	0.000	25
Image Power	0.3050	0.2966 to 0.3103	0.000	0.000	0.000	26
Laplacian Energy	0.3059	0.2986 to 0.3138	0.000	0.000	0.000	27
Mean Absolute Laplacian	0.3097	0.3011 to 0.3192	0.000	0.000	0.000	28
Mean Intensity	0.3237	0.3150 to 0.3290	0.000	0.000	0.000	29
Intensity Skewness Index	0.3433	0.3346 to 0.3469	0.000	0.000	0.000	30
Maximal Intensity	0.3462	0.3413 to 0.3513	0.000	0.000	0.000	31
GLCM Contrast	0.3704	0.3639 to 0.3751	0.000	0.000	0.000	32

https://doi.org/10.3390/jimaging12090434

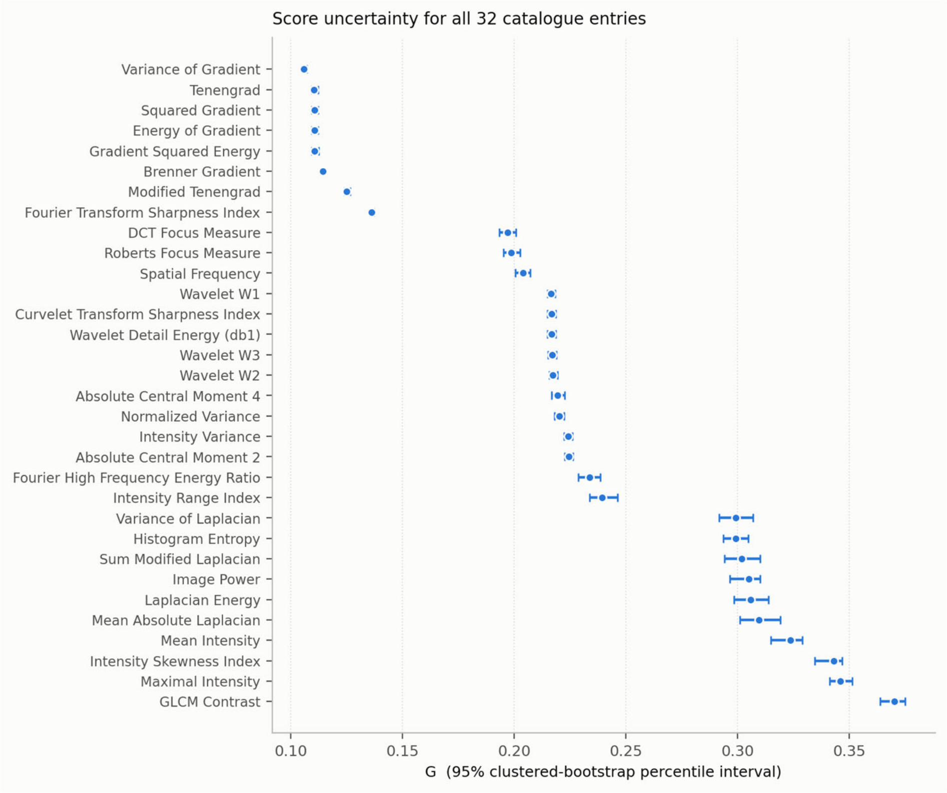

Figure S5. Clustered-bootstrap percentile intervals for G across all 32 catalogue entries, from 1000 paired replicates with the white-cell domain clustered by slide. Entries are ordered by mean G. The leading gradient entries are separated from the rest of the pool by margins far larger than their intervals, while several mid-table entries have overlapping intervals and no stable ordering between them. Intervals describe resampling stability under a fixed scoring policy and not sensitivity to the policy itself, which is shown in Figure S6.

Table S15. Criterion-weight sensitivity for all 32 entries, from 1000 draws of a flat Dirichlet distribution over the ten weights. Probabilities are the fraction of sampled weightings in which the entry attains that position. Entries with a zero rank-one probability may still appear frequently in the top three or top five. These frequencies explore scoring priorities and are not sampling confidence probabilities.

Entry	Family	P(Rank 1)	P(Top 3)	P(Top 5)
Variance of Gradient	gradient	0.679	0.826	0.853
Fourier Transform Sharpness Index	frequency	0.138	0.239	0.252
Brenner Gradient	gradient	0.044	0.137	0.157
Intensity Range Index	statistical	0.038	0.057	0.059
Intensity Skewness Index	statistical	0.029	0.043	0.049
Spatial Frequency	gradient	0.023	0.049	0.058
Maximal Intensity	statistical	0.014	0.027	0.029
Tenengrad	gradient	0.013	0.834	0.911
Normalized Variance	statistical	0.011	0.037	0.038
Fourier High Frequency Energy Ratio	frequency	0.006	0.029	0.040
GLCM Contrast	texture	0.003	0.015	0.019
Histogram Entropy	entropy	0.001	0.004	0.008
Roberts Focus Measure	gradient	0.001	0.024	0.043
Variance of Laplacian	laplacian	0.000	0.027	0.037
Sum Modified Laplacian	laplacian	0.000	0.000	0.016
Energy of Gradient	gradient	0.000	0.437	0.881

Wavelet W1	wavelet	0.000	0.006	0.012
Wavelet W2	wavelet	0.000	0.000	0.000
Wavelet W3	wavelet	0.000	0.000	0.006
Wavelet Detail Energy (db1)	wavelet	0.000	0.000	0.004
Curvelet Transform Sharpness Index	wavelet_alias	0.000	0.003	0.007
DCT Focus Measure	frequency	0.000	0.042	0.056
Squared Gradient	gradient	0.000	0.102	0.836
Gradient Squared Energy	gradient	0.000	0.000	0.522
Mean Intensity	statistical	0.000	0.004	0.006
Intensity Variance	statistical	0.000	0.000	0.007
Laplacian Energy	laplacian	0.000	0.000	0.013
Mean Absolute Laplacian	laplacian	0.000	0.011	0.027
Modified Tenengrad	gradient	0.000	0.047	0.049
Absolute Central Moment 2	statistical	0.000	0.000	0.005
Absolute Central Moment 4	statistical	0.000	0.000	0.000
Image Power	statistical	0.000	0.000	0.000

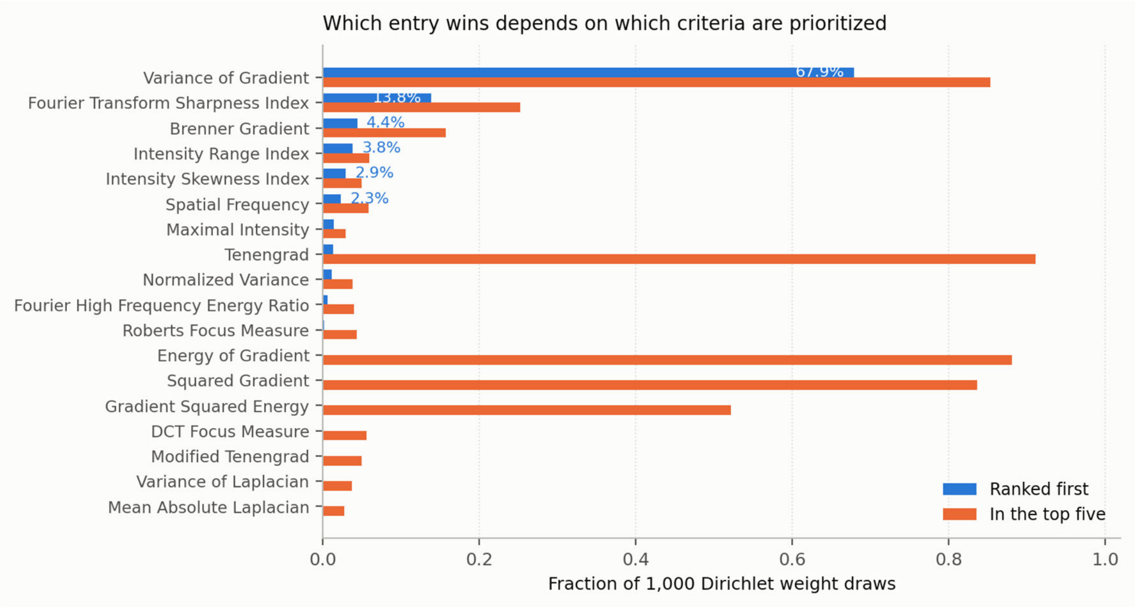

Figure S6. Criterion-weight sensitivity from 1000 draws of a flat Dirichlet distribution over the ten weights, for every entry reaching the top five in more than two per cent of draws. Variance of Gradient wins most draws but not all, and both gradient and non-gradient entries appear among the winners, so an application prioritizing different criteria may select a different operator on unchanged data. Entries that never rank first can still appear in the top five in most draws. These are frequencies over scoring policies and are not sampling confidence probabilities.

Table S16. Mean absolute displacement from REF-B under four aggregation estimands, for all 32 entries. Equal-domain macro is the primary estimand and weights each domain equally. Per-stack micro weights each stack equally and is effectively a white-cell result because that domain supplies 98.7 per cent of stacks. Balanced subsampling draws 30 stacks per domain, repeated 1000 times, and its standard deviation is across those repeats. These estimands answer different questions and are not interchangeable estimates of one population quantity.

Entry	Equal-Domain Macro	Per-Stack Micro	Balanced Mean	Balanced SD
Tenengrad	0.106	0.026	0.106	0.045
Brenner Gradient	0.072	0.080	0.074	0.036
Variance of Laplacian	2.810	0.813	2.812	0.149

Sum Modified Laplacian	2.572	0.840	2.571	0.160
Normalized Variance	0.939	2.244	0.940	0.063
Energy of Gradient	0.106	0.026	0.106	0.045
Histogram Entropy	2.966	1.999	2.967	0.126
GLCM Contrast	2.330	0.298	2.334	0.067
Variance of Gradient	0.146	0.111	0.146	0.047
Fourier Transform Sharpness Index	0.080	0.079	0.080	0.032
Fourier High Frequency Energy Ratio	1.615	0.494	1.622	0.130
Roberts Focus Measure	0.914	0.128	0.917	0.123
Wavelet W1	1.643	0.221	1.645	0.074
Wavelet W2	1.643	0.221	1.645	0.074
Wavelet W3	1.643	0.221	1.645	0.074
Wavelet Detail Energy (db1)	1.643	0.221	1.645	0.074
Curvelet Transform Sharpness Index	1.643	0.221	1.645	0.074
DCT Focus Measure	0.893	0.612	0.898	0.124
Intensity Skewness Index	5.596	2.940	5.594	0.122
Squared Gradient	0.106	0.026	0.106	0.045
Gradient Squared Energy	0.106	0.026	0.106	0.045
Mean Intensity	4.468	4.922	4.468	0.099
Intensity Variance	0.827	2.242	0.828	0.058
Intensity Range Index	1.236	1.225	1.231	0.074
Maximal Intensity	1.452	1.219	1.446	0.080
Laplacian Energy	2.810	0.813	2.812	0.149
Mean Absolute Laplacian	3.105	0.823	3.104	0.134
Spatial Frequency	1.122	0.163	1.124	0.115
Modified Tenengrad	0.112	0.038	0.112	0.047
Absolute Central Moment 2	0.827	2.242	0.828	0.058
Absolute Central Moment 4	0.881	2.073	0.881	0.061
Image Power	2.216	2.295	2.213	0.135

Table S17. Leave-one-domain-out ranks for all 32 entries. Each column gives the rank obtained when the named domain is omitted and the score is recomputed over the remaining four. This describes dependence on the five observed domains and is not an estimate of performance on an unobserved domain.

Entry	Omit WBC	Omit TBI	Omit PBS	Omit BMA	Omit TBF
Variance of Gradient	1	1	4	1	1
Tenengrad	2	2	3	2	2
Squared Gradient	3	3	5	3	3
Energy of Gradient	4	4	1	4	4
Gradient Squared Energy	5	5	2	5	5
Brenner Gradient	6	6	6	6	6
Modified Tenengrad	7	7	8	7	8
Fourier Transform Sharpness Index	8	8	7	8	10
DCT Focus Measure	9	10	9	10	11
Roberts Focus Measure	11	9	13	14	7
Spatial Frequency	15	11	15	15	9
Wavelet W1	16	12	16	16	12
Curvelet Transform Sharpness Index	17	13	18	17	14
Wavelet Detail Energy (db1)	18	14	17	18	15
Wavelet W3	19	15	19	19	13
Wavelet W2	20	16	20	20	16
Absolute Central Moment 4	14	19	10	9	19

Normalized Variance	10	18	14	11	18
Intensity Variance	12	21	11	12	20
Absolute Central Moment 2	13	22	12	13	21
Fourier High Frequency Energy Ratio	22	17	22	22	17
Intensity Range Index	21	20	21	21	22
Histogram Entropy	24	30	23	23	30
Variance of Laplacian	26	26	24	24	24
Sum Modified Laplacian	27	27	26	26	23
Laplacian Energy	28	28	25	27	25
Image Power	25	23	28	25	27
Mean Absolute Laplacian	29	25	27	28	26
Mean Intensity	30	29	29	29	29
Intensity Skewness Index	31	24	31	31	31
Maximal Intensity	23	32	30	30	32
GLCM Contrast	32	31	32	32	28

S9. Computational Cost and Image-Resampling Sensitivity

Runtime was measured on one x86-64 workstation in Python 3.12 under the recorded software environment. Three deterministic images per domain were evaluated after two warm-ups with seven repeats, and the domain, resolution and operator job order was randomized. The OMP, OpenBLAS, MKL and NumExpr thread environment variables were set to one, while the recorded OpenCV runtime reported 32 threads; the protocol is therefore not described as fully single-threaded. All timings are comparative operator costs under this protocol. They exclude exposure, transfer, stage motion, settling, readout, search and controller overhead, and no latency claim for a complete autofocus system follows from them.

Two aggregate statistics are reported for every operator because they answer different questions. The equal-domain macro statistic is the arithmetic mean of the five domain-specific median kernel times. The corpus-weighted micro statistic is the plane-count-weighted mean of those same five medians. They differ by roughly a factor of forty purely because the small 200 by 200 white-cell planes dominate the observed corpus, and both are given so that corpus composition is visible rather than hidden inside a single average.

Table S18. Median operator-kernel time per plane at native acquisition dimensions, by operator and domain, in milliseconds. Preprocessing time is reported separately in the machine-readable release, together with combined timings and the full distribution of repeats.

Entry	WBC	TBI	PBS	BMA	TBF
Variance of Gradient	0.116	37.000	37.480	35.427	35.493
Tenengrad	0.081	24.175	20.189	33.832	27.969
Squared Gradient	0.079	24.517	28.875	33.662	31.190
Energy of Gradient	0.079	23.999	31.760	30.576	30.603
Gradient Squared Energy	0.076	23.745	33.475	33.678	29.122
Brenner Gradient	0.043	13.875	13.171	14.978	13.352
Modified Tenengrad	0.786	378.511	343.217	353.478	364.927
Fourier Transform Sharpness Index	0.532	209.082	200.761	198.680	196.045
DCT Focus Measure	0.242	70.258	76.035	75.193	74.808
Roberts Focus Measure	0.082	26.939	23.078	31.935	31.929
Spatial Frequency	0.101	48.480	48.251	49.425	50.459
Wavelet W1	0.254	132.831	137.357	136.820	137.234
Curvelet Transform Sharpness Index	0.261	133.608	142.916	141.960	139.888
Wavelet Detail Energy (db1)	0.253	135.829	147.041	142.630	137.928

Wavelet W3	0.356	101.012	155.033	151.906	152.090
Wavelet W2	0.345	146.463	160.647	150.195	154.767
Absolute Central Moment 4	0.507	103.121	96.638	96.272	96.852
Normalized Variance	0.067	16.118	18.029	18.461	18.808
Intensity Variance	0.045	21.626	21.645	23.667	20.900
Absolute Central Moment 2	0.057	30.440	30.468	29.330	31.600
Fourier High Frequency Energy Ratio	0.599	221.358	213.453	209.766	210.582
Intensity Range Index	0.024	8.013	10.781	8.579	9.665
Histogram Entropy	0.168	55.416	54.798	55.069	54.214
Variance of Laplacian	0.068	15.629	18.094	16.623	14.831
Sum Modified Laplacian	0.137	36.732	32.020	31.710	32.845
Laplacian Energy	0.310	166.474	159.330	157.067	156.353
Image Power	0.027	15.393	15.444	17.511	17.485
Mean Absolute Laplacian	0.320	167.340	162.634	157.855	156.655
Mean Intensity	0.020	6.403	6.475	9.493	9.261
Intensity Skewness Index	0.349	58.240	54.850	53.180	53.750
Maximal Intensity	0.017	5.820	6.021	5.828	6.000
GLCM Contrast	9.698	1497.041	1410.164	1384.950	1412.275

Table S19. Equal-domain macro and plane-count-weighted micro kernel times per plane, in milliseconds, at three standardized image sizes and at native acquisition dimensions. Native combines the different dimensions of the five domains and is a workload category rather than a fourth numerical image size, so the values in that column are not part of a continuous size trend.

Entry	128 Macro	512 Macro	1024 Macro	native Macro	native Micro
Variance of Gradient	0.064	0.738	3.281	29.103	0.721
Tenengrad	0.041	0.587	2.344	21.249	0.527
Squared Gradient	0.042	0.541	2.514	23.664	0.591
Energy of Gradient	0.043	0.585	2.380	23.404	0.590
Gradient Squared Energy	0.042	0.575	2.432	24.019	0.586
Brenner Gradient	0.021	0.314	1.432	11.084	0.270
Modified Tenengrad	0.369	6.364	27.319	288.184	6.825
Fourier Transform Sharpness Index	0.204	3.124	19.855	161.020	3.864
DCT Focus Measure	0.098	1.476	8.029	59.307	1.498
Roberts Focus Measure	0.038	0.617	2.571	22.793	0.578
Spatial Frequency	0.041	0.811	3.621	39.343	0.936
Wavelet W1	0.135	1.937	11.962	108.899	2.555
Curvelet Transform Sharpness Index	0.135	1.956	13.206	111.727	2.622
Wavelet Detail Energy (db1)	0.134	1.970	12.940	112.736	2.615
Wavelet W3	0.204	2.445	15.010	112.079	2.864
Wavelet W2	0.179	2.355	14.728	122.483	2.950
Absolute Central Moment 4	0.188	3.104	12.533	78.678	2.134
Normalized Variance	0.040	0.365	1.740	14.296	0.376
Intensity Variance	0.030	0.402	1.647	17.577	0.406
Absolute Central Moment 2	0.028	0.528	2.339	24.379	0.578
Fourier High Frequency Energy Ratio	0.273	3.698	20.511	171.152	4.158
Intensity Range Index	0.014	0.175	0.816	7.412	0.187
Histogram Entropy	0.087	1.187	4.740	43.933	1.084
Variance of Laplacian	0.040	0.403	1.815	13.049	0.334
Sum Modified Laplacian	0.058	0.748	2.916	26.689	0.686
Laplacian Energy	0.156	2.708	10.934	127.907	2.963
Image Power	0.015	0.289	1.217	13.172	0.311
Mean Absolute Laplacian	0.158	2.698	11.712	128.961	2.992

Mean Intensity	0.013	0.150	0.652	6.330	0.162
Intensity Skewness Index	0.136	1.760	7.896	44.074	1.257
Maximal Intensity	0.011	0.146	0.659	4.737	0.117
GLCM Contrast	3.439	54.934	220.928	1142.826	33.353

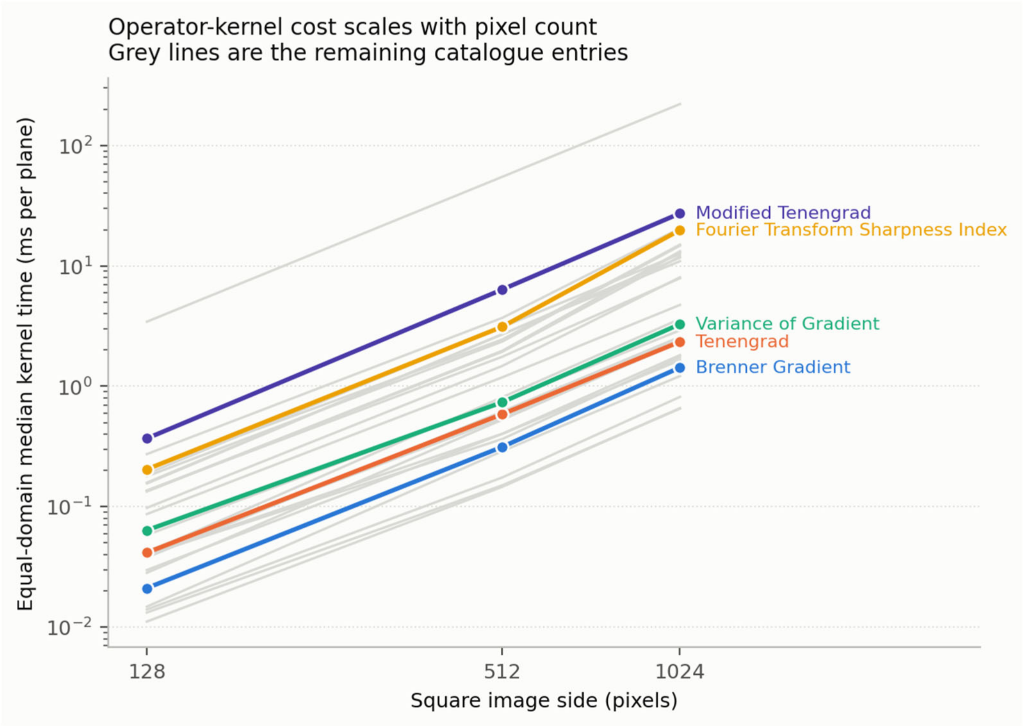

Figure S7. Equal-domain median operator-kernel time against square image side, on logarithmic axes. Five representative entries are drawn in colour and directly labelled; the remaining catalogue entries are shown in grey for context. Cost scales approximately with pixel count across the tested sizes, and the ordering of operators is stable across them, so the choice between these operators on cost grounds does not depend on the working resolution. Values are those tabulated in Table S19 and exclude acquisition and control overhead.

The image-resampling experiment is separate from the primary ranking and uses a narrower score. Fifteen stacks, contributed as the first, middle and last stack of each domain in deterministic order, were evaluated under 14 conditions that vary aspect-preserving scale, interpolation kernel and forced-square distortion separately from a common aspect-preserving base. Within the experiment a descriptive four-criterion score combining peak error, false maxima, curve roughness and full width at half maximum is used. Ranks are internal to this experiment and cannot be compared with the primary ten-criterion ranking, and the magnitude of movement here cannot be compared with the criterion-weighting analysis as a decomposition of sensitivity.

Table S20. Rank within the controlled resampling experiment for all 32 entries under five representative conditions. Ranks use the descriptive four-criterion score over the same 32-entry pool and are internal to this experiment. Only within-experiment movement is interpretable.

Entry	Native	0.5× Area	2× Area	512 sq. Area	512 sq. Nearest
Fourier Transform Sharpness Index	1	1	1	1	1
Energy of Gradient	2	14	5	9	9
Gradient Squared Energy	3	15	6	10	10
Squared Gradient	4	16	7	11	11
Tenengrad	5	17	8	12	12

Modified Tenengrad	6	19	15	13	13
Variance of Gradient	7	21	14	14	14
Brenner Gradient	8	13	12	8	8
Normalized Variance	9	23	17	22	22
Absolute Central Moment 2	10	24	18	23	23
Intensity Variance	11	25	19	24	24
Absolute Central Moment 4	12	26	20	25	25
DCT Focus Measure	13	18	4	16	16
Curvelet Transform Sharpness Index	14	3	24	2	3
Wavelet Detail Energy (db1)	15	4	25	3	4
Wavelet W1	16	5	26	4	5
Wavelet W2	17	6	27	5	6
Wavelet W3	18	7	28	6	7
Roberts Focus Measure	19	12	9	15	15
Spatial Frequency	20	2	3	7	2
Intensity Range Index	21	28	22	26	27
Histogram Entropy	22	27	23	28	28
Fourier High Frequency Energy Ratio	23	20	16	17	18
Intensity Skewness Index	24	29	29	29	29
GLCM Contrast	25	22	21	27	26
Maximal Intensity	26	30	30	30	30
Image Power	27	31	31	31	31
Mean Absolute Laplacian	28	9	2	18	17
Laplacian Energy	29	10	11	20	20
Variance of Laplacian	30	11	10	21	19
Sum Modified Laplacian	31	8	13	19	21
Mean Intensity	32	32	32	32	32

https://doi.org/10.3390/jimaging12090434

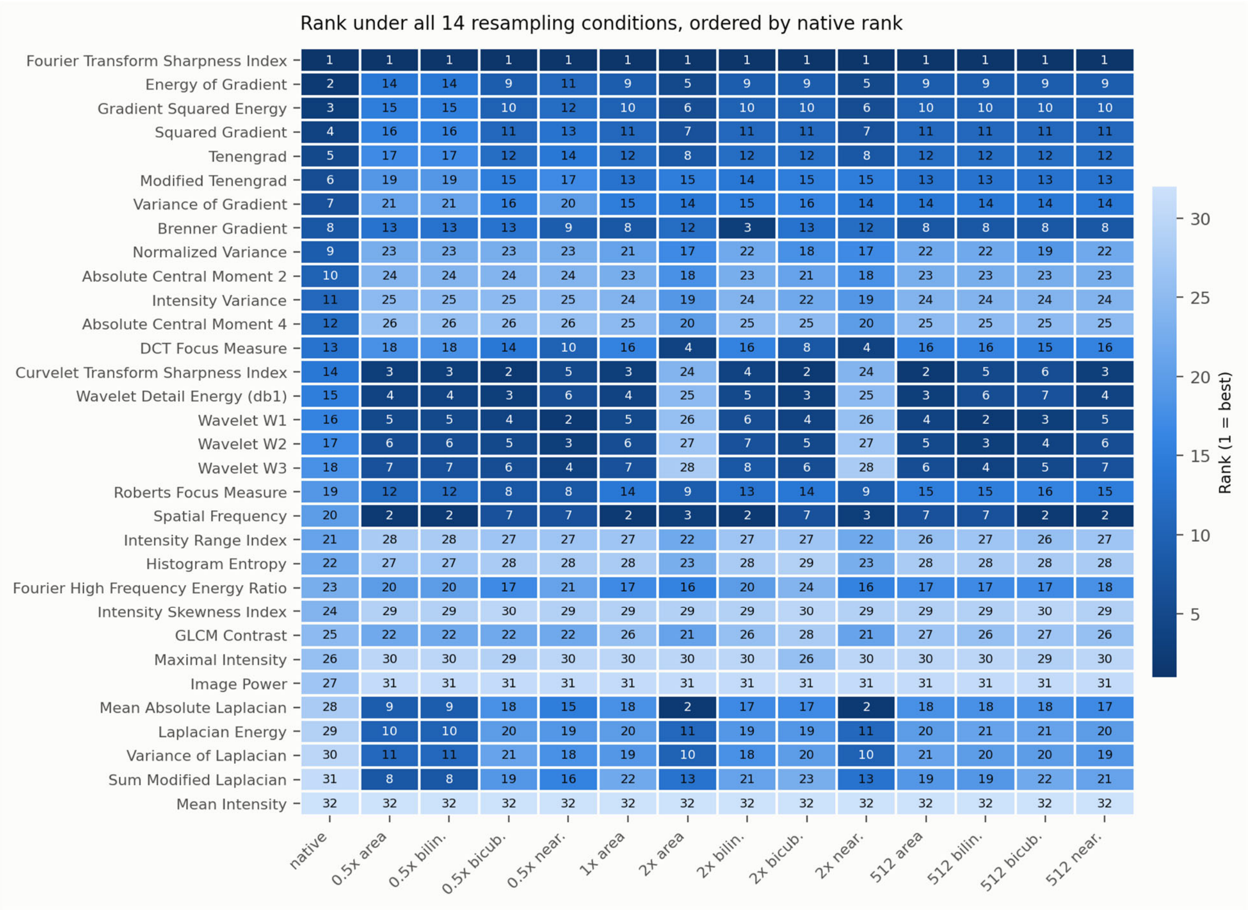

Figure S8. Rank of every catalogue entry under all 14 conditions of the controlled resampling experiment, ordered by rank at native acquisition dimensions. Ranks use the descriptive four-criterion score over the same 32-entry pool and are internal to this experiment. Two patterns are visible: the wavelet-labelled and Laplacian entries improve sharply under downsampling while the gradient entries lose ground, and only two entries hold a single rank across all 14 conditions, the Fourier Transform Sharpness Index at rank one and Mean Intensity at rank 32. Only within-experiment movement is interpretable.

Table S21. Descriptive associations between rank movement and the change in each contributing descriptor, by resampling condition. Spearman rho is computed across the 32 entries. The p values are descriptive only. These descriptors also contribute to the four-criterion score used to define the ranks, so each association contains a part-whole relationship and none is evidence of mechanism.

Condition	Association	Spearman Rho	p (Descriptive)	n
aspect 0.5× area	mean noise level reduction	0.104	0.572	32
aspect 0.5× area	mean false maxima count reduction	0.582	0.000	32
aspect 0.5× area	mean absolute peak error slices reduction	0.143	0.433	32
aspect 0.5× area	mean fwhm slices reduction	0.526	0.002	32
aspect 0.5× bicubic	mean noise level reduction	0.118	0.520	32
aspect 0.5× bicubic	mean false maxima count reduction	0.364	0.041	32
aspect 0.5× bicubic	mean absolute peak error slices reduction	−0.135	0.461	32
aspect 0.5× bicubic	mean fwhm slices reduction	0.125	0.497	32
aspect 0.5× bilinear	mean noise level reduction	0.104	0.572	32
aspect 0.5× bilinear	mean false maxima count reduction	0.582	0.000	32
aspect 0.5× bilinear	mean absolute peak error slices reduction	0.143	0.433	32
aspect 0.5× bilinear	mean fwhm slices reduction	0.526	0.002	32
aspect 0.5× nearest	mean noise level reduction	−0.252	0.165	32

aspect 0.5× nearest	mean false maxima count reduction	0.483	0.005	32
aspect 0.5× nearest	mean absolute peak error slices reduction	−0.029	0.874	32
aspect 0.5× nearest	mean fwhm slices reduction	0.477	0.006	32
aspect 2× area	mean noise level reduction	−0.083	0.651	32
aspect 2× area	mean false maxima count reduction	0.141	0.440	32
aspect 2× area	mean absolute peak error slices reduction	0.676	0.000	32
aspect 2× area	mean fwhm slices reduction	0.124	0.498	32
aspect 2× bicubic	mean noise level reduction	0.394	0.026	32
aspect 2× bicubic	mean false maxima count reduction	0.385	0.030	32
aspect 2× bicubic	mean absolute peak error slices reduction	−0.150	0.413	32
aspect 2× bicubic	mean fwhm slices reduction	−0.429	0.014	32
aspect 2× bilinear	mean noise level reduction	−0.100	0.585	32
aspect 2× bilinear	mean false maxima count reduction	−0.149	0.417	32
aspect 2× bilinear	mean absolute peak error slices reduction	0.459	0.008	32
aspect 2× bilinear	mean fwhm slices reduction	0.292	0.105	32
aspect 2× nearest	mean noise level reduction	−0.083	0.651	32
aspect 2× nearest	mean false maxima count reduction	0.141	0.440	32
aspect 2× nearest	mean absolute peak error slices reduction	0.676	0.000	32
aspect 2× nearest	mean fwhm slices reduction	0.124	0.498	32
square 512 area	mean noise level reduction	0.123	0.501	32
square 512 area	mean false maxima count reduction	0.585	0.000	32
square 512 area	mean absolute peak error slices reduction	0.033	0.858	32
square 512 area	mean fwhm slices reduction	0.083	0.650	32
square 512 bicubic	mean noise level reduction	0.086	0.640	32
square 512 bicubic	mean false maxima count reduction	0.261	0.148	32
square 512 bicubic	mean absolute peak error slices reduction	−0.304	0.090	32
square 512 bicubic	mean fwhm slices reduction	0.290	0.108	32
square 512 bilinear	mean noise level reduction	−0.119	0.518	32
square 512 bilinear	mean false maxima count reduction	0.258	0.154	32
square 512 bilinear	mean absolute peak error slices reduction	−0.054	0.771	32
square 512 bilinear	mean fwhm slices reduction	0.234	0.198	32
square 512 nearest	mean noise level reduction	0.282	0.118	32
square 512 nearest	mean false maxima count reduction	0.577	0.001	32
square 512 nearest	mean absolute peak error slices reduction	0.000	1.000	32
square 512 nearest	mean fwhm slices reduction	−0.143	0.434	32

S10. Symbolic-Composite Provenance and Machine-Readable Inventory

The symbolic-composition analysis is exploratory and does not enter the primary ranking. Strongly typed genetic programming was run in five leave-one-domain-out folds and one all-domain refit. In each fold, ten terminals were selected using training-domain results only, with family stratification and at most two terminals per family. Ten seeds used population 500, 100 generations, protected arithmetic primitives, maximum depth 10 and maximum size 35 nodes.

Retention proceeded in a fixed order. Exact duplicates were collapsed, same-fold representatives were deduplicated by correlation of their mean held-out curves, and the survivors were ordered by held-out score, complexity and seed. Fourteen composites were retained. Evolution used only training domains, but this post-evolution processing was not fully held-out: held-out responses informed representative selection and ordering. The fold scores are therefore descriptive transfer summaries and not unbiased validation estimates. Even under that favourable selection, no retained composite improved on the

strongest single operator in the matched full-data union pool, where the best composite reached G = 0.1622 at rank 10 against G = 0.1182 at rank 1 for Variance of Gradient.

Table S22. Provenance of the 14 retained symbolic composites. Fold identifies the leave-one-domain-out fold or the all-domain refit; the held-out score is undefined for the refit because no domain is held out. The final score is computed in the matched full-data union pool that contains both the composites and the single operators, because the normalization range depends on the candidates present. Node count and depth are measured on the exact retained expression. Full expressions are released in machine-readable form.

ID	Fold	Seed	Training Score	Held-Out Score	Final Score	Nodes	Depth	Retention Reason
CFM1	FINAL_ALL	44	0.1799	not applicable	0.1622	33	7	final all-domain refit
CFM2	WBC	45	0.1846	0.1692	0.1633	28	6	best representative after exact-expression and within-fold functional deduplication at correlation ≥ 0.99
CFM3	WBC	48	0.1837	0.1950	0.1634	33	8	best representative after exact-expression and within-fold functional deduplication at correlation ≥ 0.99
CFM4	TBF	49	0.1796	0.2357	0.1623	9	5	best representative after exact-expression and within-fold functional deduplication at correlation ≥ 0.99
CFM5	TBF	51	0.1856	0.2363	0.1672	18	7	best representative after exact-expression and within-fold functional deduplication at correlation ≥ 0.99
CFM6	TBF	48	0.1840	0.2367	0.1695	24	8	best representative after exact-expression and within-fold functional deduplication at correlation ≥ 0.99
CFM7	TBF	43	0.1857	0.2518	0.1648	14	6	best representative after exact-expression and within-fold functional deduplication at correlation ≥ 0.99
CFM8	TBI	50	0.1802	0.2688	0.1668	13	6	best representative after exact-expression and within-fold functional deduplication at correlation ≥ 0.99
CFM9	BMA	51	0.1782	0.2769	0.1668	13	5	best representative after exact-expression and within-fold functional deduplication at correlation ≥ 0.99
CFM10	BMA	48	0.1786	0.2851	0.1675	18	8	best representative after exact-expression and within-fold functional deduplication at correlation ≥ 0.99
CFM11	PBS	43	0.1782	0.2957	0.1704	11	4	best representative after exact-expression and within-fold

								functional deduplication at correlation ≥ 0.99
CFM12	PBS	49	0.1770	0.3056	0.1721	25	10	best representative after exact-expression and within-fold functional deduplication at correlation ≥ 0.99
CFM13	TBI	48	0.1687	0.3145	0.1728	27	10	best representative after exact-expression and within-fold functional deduplication at correlation ≥ 0.99
CFM14	TBI	45	0.1787	0.3222	0.1792	11	4	best representative after exact-expression and within-fold functional deduplication at correlation ≥ 0.99

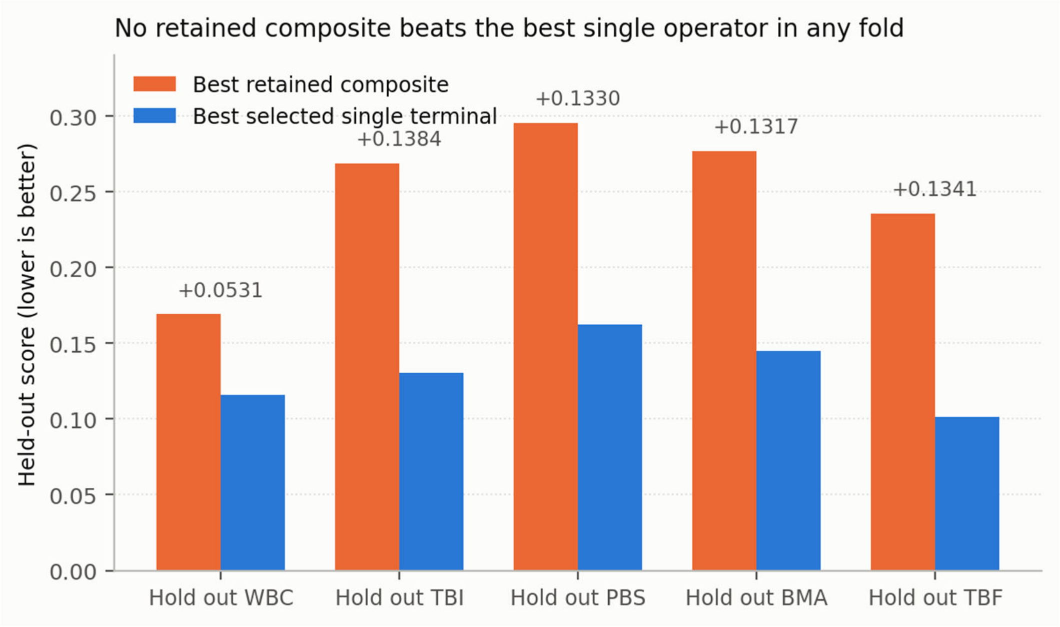

Figure S9. Best retained symbolic composite against the best selected single terminal on each held-out domain. The composite score exceeds the single-terminal score in every fold, by 0.053 to 0.138, and lower is better, so no retained composite transfers better than the strongest single operator available to that fold. Because held-out responses informed post-evolution deduplication and ordering, these values favour the composites; the negative result holds even under that favourable selection.

Table S23. Inventory of the machine-readable release, grouped by the analysis it supports. Every value reproduced in this document is derived from these files. The release is distributed with the article and from the project repository under the MIT licence.

Group	Files	Supports
Library and provenance	operator_registry_32.json; corrected_raw_criteria.csv	S1, S2
Encoding range and binning	corrected_entropy_rrmse_per_stack.csv; submitted_vs_corrected_domain_summary.csv; submitted_vs_corrected_change_impact.csv	S3
Criteria, weights and policy	deterministic_weight_and_alpha_sensitivity.csv; weight_provenance.md; dirichlet_rank_frequencies.csv	S4, S8
Reference constructions	reference_agreement_by_domain.csv; reference_shift_distributions.csv; reference_transition_matrices.csv; reference_provenance.csv; reference_ladder_configuration.json	S5

https://doi.org/10.3390/jimaging12090434

Rankings and scores	corrected_final_rankings.csv; corrected_domain_scores.csv; all_operators_domains_criteria_long.csv and .xlsx	S6, S7
Localization	operator_localization_axial_summary.csv; per_stack_localization.csv; per_stack_axial_localization.csv; curve_width_physical_units.csv	S7
Sampling uncertainty	rank_frequencies.csv; paired_difference_intervals.csv; wbc_slide_cluster_vs_stack_bootstrap.csv; wbc_stack_to_slide.csv; bootstrap_configuration.json; sigma_stability.csv	S8
Aggregation estimands	aggregation_estimands.csv; balanced_subsampling_rank_stability.csv; lodo_rankings.csv; domain_leave_one_out_effects.csv	S8
Computational cost	corrected_runtime_per_measure_domain.csv; corrected_runtime_per_resolution.csv; runtime_macro_micro_comparison.csv; runtime_environment.json; runtime_protocol.json	S9
Image resampling	resampling_rank_shifts.csv; resampling_operator_summary.csv; resampling_mechanism_correlations.csv; roberts_brenner_mechanism.csv; resampling_configuration.json	S9
Symbolic composites	retained_composite_provenance.csv; symbolic_audit_configuration.json	S10
Acquisition and licensing	acquisition_metadata_verified.csv; source_manifest.csv; licence_manifest.csv; figure_manifest.csv; figure_selection_manifest.csv	S1, S5

Note on scope. Every quantity in this document describes agreement with a declared algorithmic consensus, the behaviour of a focus curve, or a measured computational cost. None of the three reference constructions is optical ground truth, and no value here should be read as accuracy against a physically determined best-focus plane.

https://doi.org/10.3390/jimaging12090434
